# Advances in Smart Photovoltaic Textiles

**DOI:** 10.1021/acsnano.3c10033

**Published:** 2024-01-23

**Authors:** Iftikhar Ali, Md Rashedul Islam, Junyi Yin, Stephen J. Eichhorn, Jun Chen, Nazmul Karim, Shaila Afroj

**Affiliations:** † Centre for Print Research (CFPR), The University of the West of England, Frenchay Campus, Bristol BS16 1QY, U.K.; ‡ Department of Bioengineering, University of California, Los Angeles, Los Angeles, California 90095, United States; § Bristol Composites Institute, School of Civil, Aerospace, and Design Engineering, The University of Bristol, University Walk, Bristol BS8 1TR, U.K.; ⊥ Nottingham School of Art and Design, Nottingham Trent University, Shakespeare Street, Nottingham NG1 4GG, U.K.

**Keywords:** energy harvesting, smart textiles, wearable electronics, photovoltaic textiles, electronic textiles, solar cells, green energy, solar energy

## Abstract

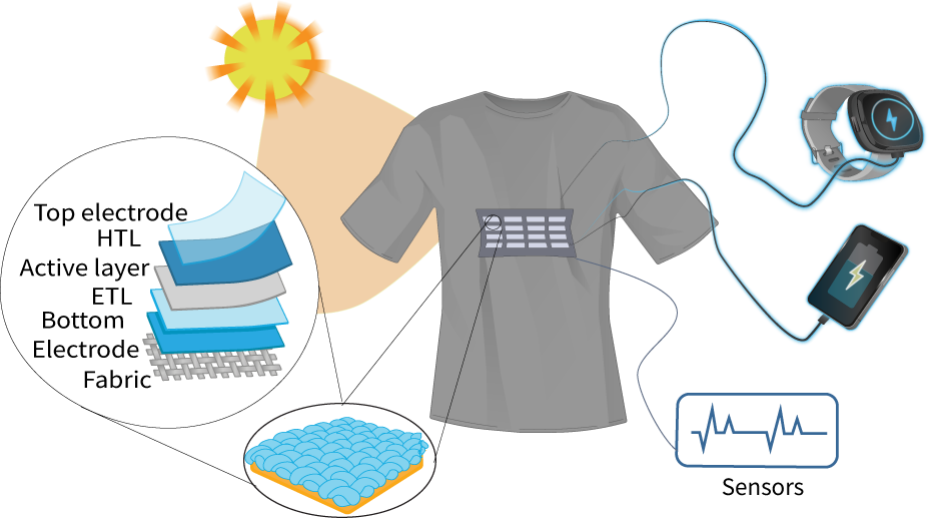

Energy harvesting textiles have emerged as a promising solution to sustainably power wearable electronics. Textile-based solar cells (SCs) interconnected with on-body electronics have emerged to meet such needs. These technologies are lightweight, flexible, and easy to transport while leveraging the abundant natural sunlight in an eco-friendly way. In this Review, we comprehensively explore the working mechanisms, diverse types, and advanced fabrication strategies of photovoltaic textiles. Furthermore, we provide a detailed analysis of the recent progress made in various types of photovoltaic textiles, emphasizing their electrochemical performance. The focal point of this review centers on smart photovoltaic textiles for wearable electronic applications. Finally, we offer insights and perspectives on potential solutions to overcome the existing limitations of textile-based photovoltaics to promote their industrial commercialization.

Wearable electronic textiles (e-textiles) have been a focus of research interest in sportswear, military uniforms, safety instruments, and healthcare applications as lightweight and portable devices to monitor vital health parameters.^1,2^ E-textiles inherit the advantages of being lightweight, flexible, wearable, and air-permeable while possessing several electronic functions.^3−7^ Functionalities such as sensing, computation, display, and communication,^8−11^ in e-textiles could facilitate the manufacturing of highly innovative and intelligent garments, which can seamlessly integrate all the sensors, actuators, energy harvesters, and energy storage components.^12−15^ However, to realize the functionalities of intelligent garments requires a lightweight, flexible, and high-performance power supply.^16−19^ Traditional power-supply technologies (e.g., batteries) are incompatible with such smart textile systems due to their bulky size, rigidity, limited lifetime, repeated replacement, release of heat during discharging, and particularly the inclusion of some toxic materials which can cause serious skin issues.^20−22^ Therefore, the key focus in powering wearable electronics is moving away from traditional battery systems to safe, lightweight, flexible, wearable counterparts, and energy-harvesting textiles are a compelling solution toward that effort.^23,24^

The need for electrical energy in human civilization is ubiquitous, and it has been expanding at a rapid pace with technological advancements.^25,26^ Traditional methods for electricity generation, such as the burning of fossil fuels, are nearing the end of their residual value and are expected to be depleted within the next 150 years.^25^ A vast quantity of hazardous pollutants are being released into the air during the burning of fossil fuels, causing a wide range of public health concerns, such as cancer,^27^ eye illness,^28^ and respiratory disorders.^29,30^ In addition, the release of excess carbon dioxide (CO_2_) and other greenhouse gases from the combustion of fossil fuels also shows a serious negative impact on climate change and human sustainability.^31,32^ Considering the rapidly growing need for nonfossil fuel sources of energy and the mounting environmental challenges, the development of sustainable and renewable energy alternatives has become a priority to build a sustainable environment for human civilization.^33,34^ Among renewable energy choices, sunlight is one of the most abundant, green, and high energy density sources. A report from the Massachusetts Institute of Technology elaborated that the earth is constantly being bombarded by ∼173,000 terawatts (trillions of watts) of solar energy, equivalent to producing more than ten thousand times the amount of energy used per year globally.^35^ Although technologies for solar energy harvesting, such as silicon-based SCs,^36,37^ are already established and being used in various solar power plants. Though the current state of flexible and wearable SCs falls short of the efficiency and durability required to effectively compete with traditional energy generation technologies in terms of power generation capabilities.^38^ However, flexible, and wearable electrodes and/or substrate materials in SCs provide structural flexibility, which are highly attractive for a large number of emerging portable and lightweight consumer devices.^39,40^ Flexible plastic, elastomeric and textile substrates possess better biocompatibility, stretchability, transparency, and wearability.^41^ In addition, comfortability, and ability to integrate with other electrical components makes textile-based SCs a suitable candidate for the next generation of self-powered wearable e-textile applications, including for personalized healthcare. Figure 1 illustrates the basic concept of a wearable photovoltaic textile garment for powering wearable and portable devices.

**Figure 1:**
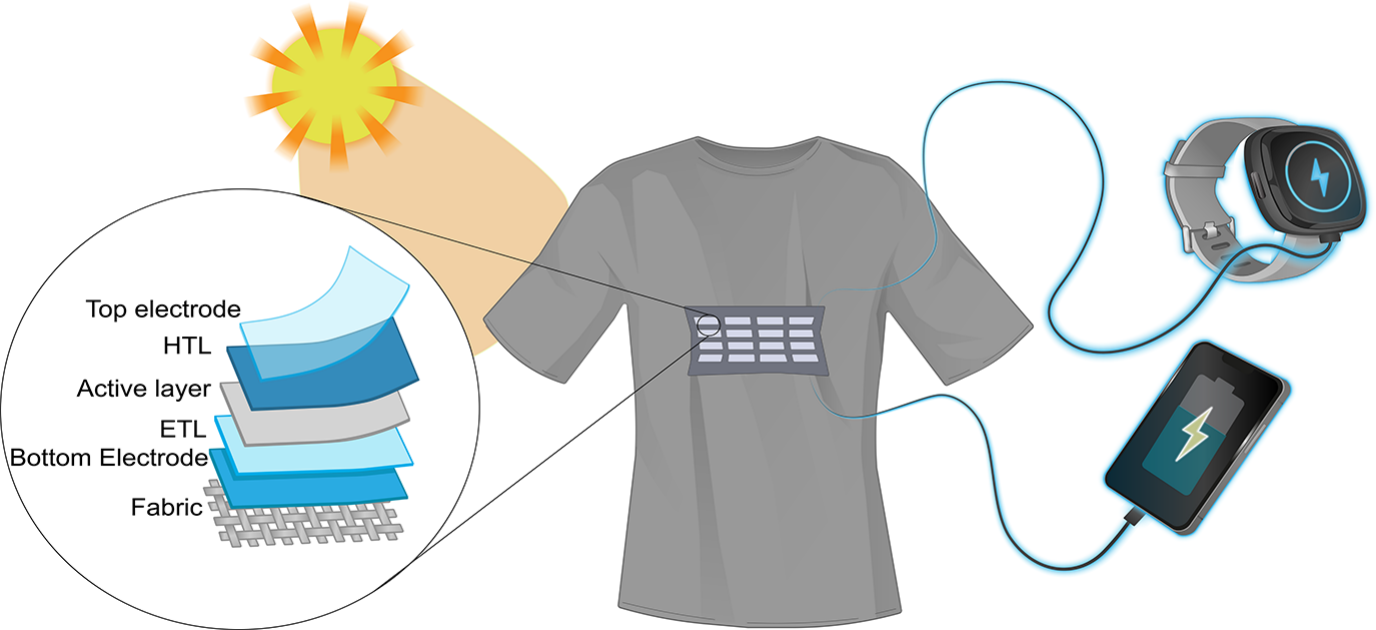
Textile solar cells for powering wearable and portable devices.

Considering the potential of smart solar textiles for the next generation of wearable power supply, this Review specifically focuses on smart textiles for solar energy harvesting as a wearable and sustainable power-supply system. We begin our review by introducing various energy harvesting approaches and their elemental categories. We then discuss SCs for energy harvesting and several generations of development. In each case, we summarize their construction and working principles. In the following section, we discuss the structure of textile-based SCs. The key metrics for evaluating SCs are then introduced. The different materials required for preparing several component layers of the SCs are then reviewed. The preparation of active materials and the fabrication strategies for different forms of textile-based SCs are then discussed. We subsequently summarize the electrochemical performance of the existing SCs along with their various wearability properties. Finally, we conclude our review with recommendations for future research directions in the field of textile-based SCs.

## Renewable Energy Harvesters

Energy is the essential necessity for any functionality.^42^ Since ancient times, energy has been transformed from one form to another based on the application, such as a steam engine where thermal energy is transformed into mechanical energy.^43^ In this context, energy harvesting refers to the process of transforming different sources of energy into electrical energy. Renewable energy harvesting is the practice of harnessing renewable energy sources (e.g., sunlight, wind, ambient thermal energy) to generate electricity.^44^ Experts in the field of energy harvesting are exploring various options to harvest energy by using advanced nanotechnologies.^45,46^ The most common and emerging energy harvesting technologies include nanogenerators (NGs), photovoltaic systems, electromagnetic generators, magnetoelastic generators (MEGs), and catalytic energy harvesting systems.^47,48^

There are different types of modern techniques developed to harvest ambient renewable energy, including triboelectricnanogenerators, piezoelectric nanogenerators, and magnetoelastic generators for mechanical energy conversion, pyroelectric generators (PEG) and thermoelectric generators (TEG) for harvesting ambient thermal energy .^49,50^ Especially, these modern mechanical energy harvesting techniques are increasingly gaining popularity due to their ability to harvest a variety of energy forms from the environment including human motions (walking,^51^ breathing,^52^ heartbeat pulse,^53^ etc.), vibration, flowing water,^53^ raindrops,^54^ wind,^55^ and waste heat.^56−59^

**Figure 2:**
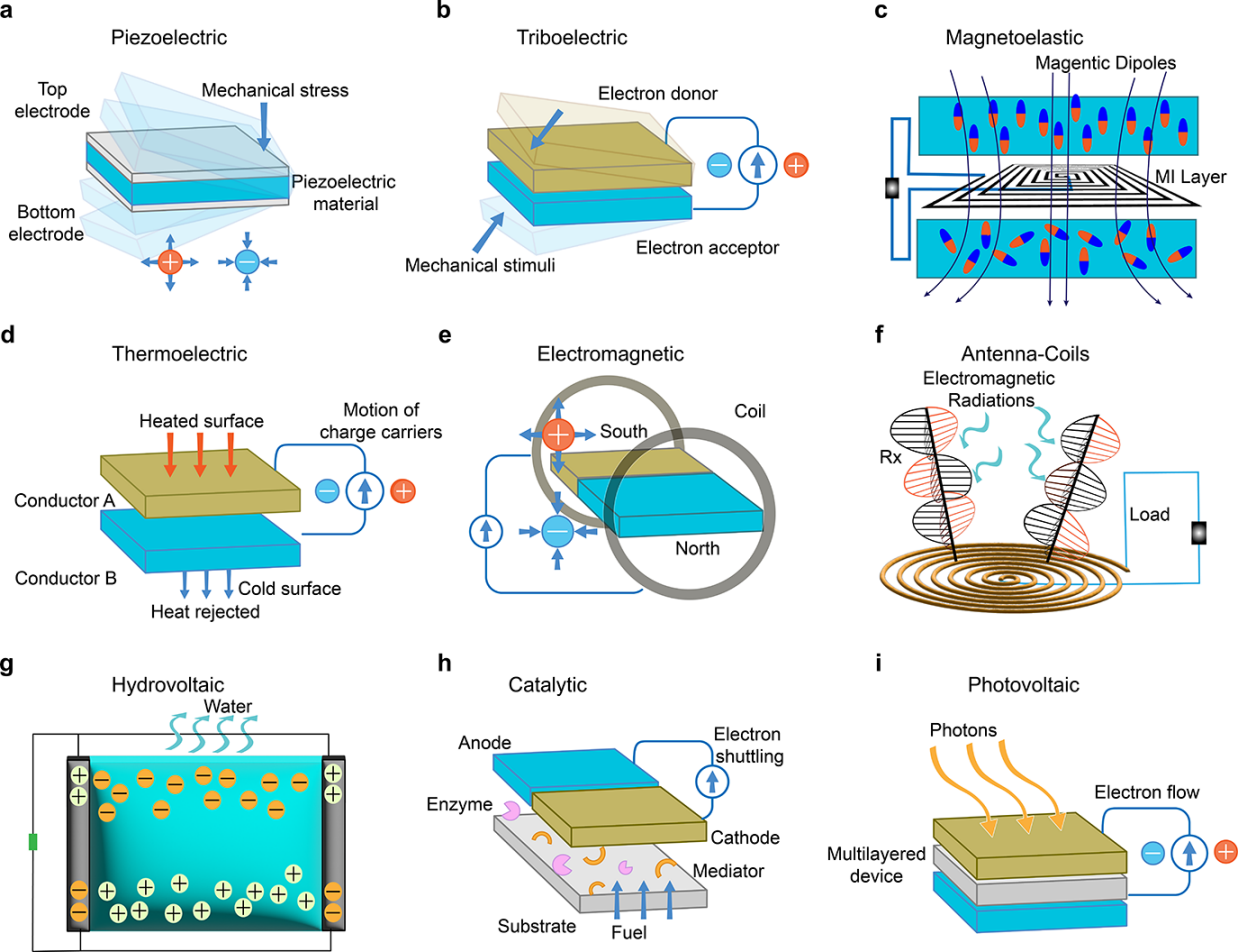
Schematics of different energy harvesters’ working mechanisms. (a) Piezoelectric, (b) triboelectric, (c) magnetoelastic, (d) thermoelectric, (e) electromagnetic, (f) antenna-coils, (g) hydrovoltaic, (h) catalytic, and (i) photovoltaic.

Nanogenerators based on the piezoelectric effect are known as piezoelectric nanogenerators (PENGs), which were introduced in 2006 by Wang et al.^60,61^ PENGs convert mechanical energy into electrical energy. The structure of a PENG device is generally a sandwich type, where two electrodes sandwich piezoelectric materials, (Figure 2a). When an external strain is applied to the two electrodes, a piezo potential difference arises between the contacts, enabling the flow of charges toward an external load.^62−64^ PENGs are widely used for textile-based energy harvesting due to their simple and straightforward mechanisms, as well as their compatibility with wearable devices.^65−68^

The Triboelectric Nanogenerator (TENG) is a device that harvests ambient mechanical energy by combining the triboelectric effect with electrostatic induction (Figure 2b).^69^ TENGs operate in four different modes: single electrode mode,^70^ contact separation mode,^71^ linear sliding mode,^72^ and free-standing mode.^73^ In all of these modes, two separate triboelectric materials, electrode connections, and an insulating layer between them are essential components.^74^ TENGs are considered the most promising choice for textile-based energy harvesters due to their high electrical output potential, flexible structure, easy and low-cost fabrication approach. Textile-based TENGs have recently been developed and fabricated on a large scale.^75^ A liquid pumping method was employed, utilizing a thin hollow polymer fiber with a liquid metal/polymer core–shell structure (LCFs). Once the LCFs were fabricated, a basic weaving machine was used to construct a textile-based TENG. These textile-based TENGs have also been utilized for energy harvesting from human movements.^76^ Silver conductive yarns wrapped in polytetrafluoroethylene (PTFE) and Nylon 66 were used, resulting in peak power densities of up to 1484 W/m^2^ in the stretching motion mode and 7531 W/m^2^ in the compressive motion mode.

To harvest the ambient biomechanical energy, the magnetoelastic generators emerges as a compelling platform technology in 2021 with intrinsic waterproofness and high current output (Figure 2c). The magnetoelastic effect, also called the Villari effect and discovered by Italian physicist Emilio Villari in 1865, is the variation of the magnetic field of a material under mechanical stress. This effect has been traditionally observed in rigid alloys with an externally applied magnetic field and has been overlooked in the field of soft bioelectronics for three reasons. First, the magnetization variation in the biomechanical stress range is limited. Second, the requirement of the external magnetic field induces structural complexity and bulkiness. Finally, there exists a large mismatch in mechanical modulus (6 orders of magnitude difference) between the magnetic alloy and human tissue. In 2021, the giant magnetoelastic effect was discovered in a soft polymer system.^77^ The giant magnetoelastic effect was further coupled with magnetic induction to invent a soft magnetoelastic generator as a fundamentally new and efficient platform technology that can convert tiny biomechanical pressure, such as arterial vibrations, into electrical signals with high fidelity.^78−82^ It features high current, low internal impedance, high stability, and decent biocompatibility, which would also revive the biomechanical energy conversion community that is currently challenged by low current, high internal impedance, and instability due to the vulnerability to water/humidity.^83^ More importantly, the soft MEGs are intrinsically waterproof since the magnetic fields can penetrate water with negligible intensity loss. Thus, they demonstrate stable performance as wearable and implantable power sources without the need of an encapsulation layer.^84−87^ This could be essentially compelling since the working environment of a bioelectronic device holds high humidity, no matter if they are skin-interfaced devices or in an implanted format.

Thermal energy is abundant in our environment, particularly in factories and manufacturing plants where it plays a critical role in daily operations. Renewable energy researchers aim to harness ambient temperatures, especially the heat generated by the human body and the surroundings.^88,89^ To harness ambient thermal energy, PEGs are one of the widely utilized technologies. These PEGs are based on the pyroelectric effect, which is the property of certain anisotropic materials in which polarization changes with the changing temperature. Nonuniform heating causes nonuniform stresses, leading to a change in the polarization through a piezoelectric action, resulting in pyroelectricity.^42,90^ The spontaneous polarization of pyroelectric materials decreases as the temperature rises and vice versa. Consequently, temperature fluctuations generate an alternating current, which is collected by electrodes.^91^ PEGs were introduced by the Wang group in 2012.^92^ On the other hand, the ambient thermal energy can be harvested by employing TEGs, which are based on the thermoelectric effect, also known as the Seebeck effect. This effect is the process by which an electric voltage is produced from a temperature gradient using a thermocouple.^93,94^ As shown in Figure 2d, a typical thermoelectric device consists of two thermoelectric layers, with one surface being heated and the other surface being cold to establish a temperature differential.^95^ A thermoelectric fabric has been developed using electrospinning and spraying techniques.^96^ To make these fabrics, carbon nanotubes (CNTs), polyvinylpyrrolidone (PVP) and polyurethane (PU) were synthesized, demonstrating stretchability of up to 250% with high air permeability. Additionally, a maximum current of 0.75 mV was generated at ambient temperature. Five devices were connected in series and fixed on a human arm to track real-time human respiration using this self-powered thermal sensor.

Electricity generation from electromagnetic radiation is one of the oldest power generation mechanisms, dating back to 1813. Faraday discovered that a varying magnetic field stimulates an electric current in a nearby circuit, providing evidence that mechanical energy can be transformed into electric energy.^97−99^ Generally, there are two basic components required to build an electromagnetic generator: a circuit/coil and a moveable magnet (Figure 2e). Electromagnetism has been explored for powering wearable electronics, particularly in shoes.^100−105^ Recently, textile-based electromagnetic energy harvesters have gained interest due to the rapid development in portable and wearable energy harvesting devices. A wearable electromagnetic generator has been developed by harnessing the ambient kinetic energy of a human hand swing motion utilizing a conductive yarn coil.^106^ However, most of the reported textile-based electromagnetic devices are heavy, inflexible, incredibly hard, and bulky, which limits their further exploration as textile-based energy harvesters.^107−109^

Electromagnetic radiation may be scavenged by antennas and coils, which has recently shown substantial potential as an alternative energy source, (Figure 2f).^110^ This technology is capable of harnessing and transforming electromagnetic energy into electrical energy.^111,112^ A wearable energy harvesting device, for example, has recently been developed with MXene (Ti_3_C_2_Tx) 5G antenna technology. The device serves as a remote and battery-less power source, allowing constant monitoring and data transfer. The antenna efficiently absorbs RF electromagnetic energy at astonishingly low input levels, 16 times lower than the threshold for standard copper antennas, while operating within the 915 MHz 5 GHz frequency range. Furthermore, the device has demonstrated exceptional mechanical flexibility, retaining over 99% power transmission efficiency despite being at a tilting angle of 90°.^113^

A Hydrovoltaic Energy Generator (HEG) is another emerging device, effectively utilizing the physiochemical properties of water to generate electricity, (Figure 2g). With the capacity to charge wearable devices continuously with DC power from an endless natural source, such as ambient humidity, HEGs are becoming increasingly popular in research and academia.^114^ A group of researchers recently developed a HEG using the ionic polymer nafion and a poly(*N*-isopropylacrylamide) hydrogel. The developed device yielded an extraordinarily high voltage of −1.86 V utilizing a single module.^115^ This cutting-edge development has the potential to significantly broaden the possibilities for wearable technology’s use of effective clean power sources.

Catalytic energy harvesters, also known as biofuel cells (Figure 2h), utilize enzymes as catalysts to transform chemical energy into electrical energy.^116^ The electrochemical oxidation of ioenzymes such as glucose and lactate, among others, at the anode and the reduction of oxygen at the cathode of a biofuel cell generate electrical energy. The advantages of such energy harvesters are their compatibility with biological environments and their ability to use biological materials as a source of energy.^117−119^ Most of the research conducted on biofuel cells has resulted in relatively small-sized devices, yielding outputs recorded at the microscale.^120,121^ For instance, the high concentration of lactate dehydrogenase found in human body sweat makes it an effective enzyme for oxidizing lactate. As a result, catalytic energy harvesters are receiving significant attention as a source for self-powered textile-based wearable devices.^116,122^

Among those already mentioned ambient energy resources, the sun is by far the most powerful and abundant resource for renewable energy.^123−125^ SCs are energy harvesting devices that absorb photons from sunlight and generate electrical energy, (Figure 2i). SCs based on silicon are known as first-generation SCs, which are not suitable for flexible electronics due to their bulky and brittle nature.^126,127^ However, second-generation and third-generation SCs (discussed in detail in later sections) have enabled the development of flexible SCs. These emerging photovoltaic technologies demonstrate the potential of implementing and harnessing energy from textile-based SCs while maintaining the comfortability required to preserve the features of clothing.^128,129^ Potential techniques for integrating SCs into textiles include fabricating SCs thin films on flexible substrates and adhering them to the textile, or directly developing SCs thin films on the fabric surface using solution-processable techniques such as printing and coating. Other methods are to build/incorporate SCs fibers/yarns/filaments into the textile’s structure. Technologies based on polymer-based and dye-sensitized SCs have gained attention due to significant improvements in conversion efficiencies.^109,130−132^ The urgent need for developing these emerging photovoltaic technologies for wearable applications has piqued interest in understanding, exploring, and garnering solutions to overcome current limitations for advancing textile-based SCs.

## Photovoltaic Energy Harvesting: Solar Cell

**Figure 3:**
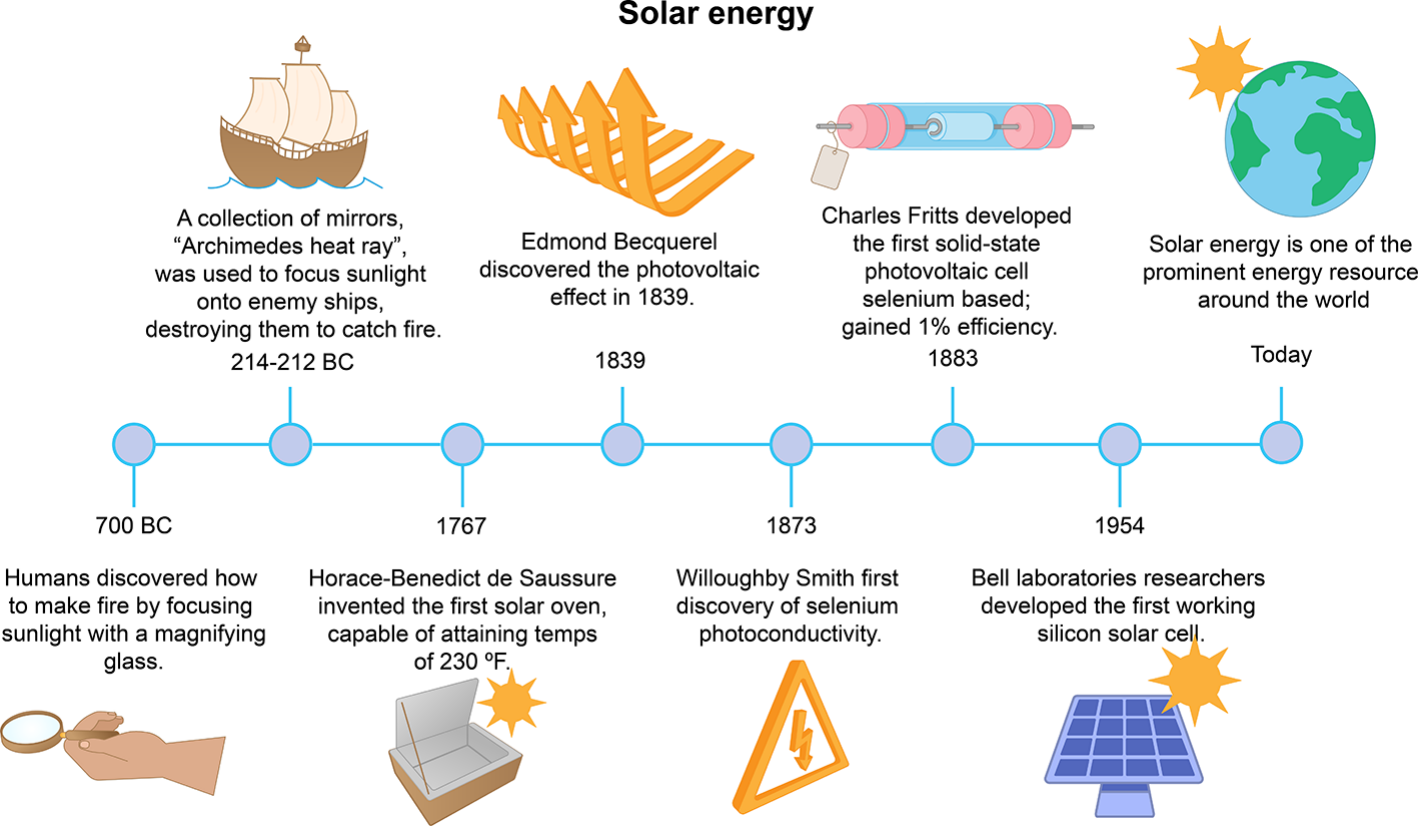
Timeline of solar energy toward the development of a practical photovoltaic system.

SCs have a long history, dating back to the early discovery of the photovoltaic effect in 1839.^133^ Alexandre-Edmond Becquerel, Antoine Cesar Becquerel, and Henri Becquerel observed tiny electric currents when they exposed metals to light while working with metallic electrodes in a liquid electrolyte. However, they could not fully explain the phenomenon at that time. Later in 1873, Willoughby Smith, discovered the impact of sunshine on selenium and its photoconductivity while working on telegraph cable materials.^134,135^ Charles Fritts, an American inventor, introduced the SC in 1883 by sandwiching the selenium (Se) between two metallic electrodes. However, those SCs were only 1% effective at converting sunlight into electrical energy and were impractical at that time.^136^ In 1954, Bell Laboratories introduced a functional silicon-based SC, marking a turning point in photovoltaics. To this day, silicon based SCs account for more than 90% of global solar energy harvesting panels.^137^Figure 3 illustrates a brief history of the utilization of solar energy in the development of the practical SCs and the scope of solar energy in today’s world.

### Construction/Working of SC

**Figure 4:**
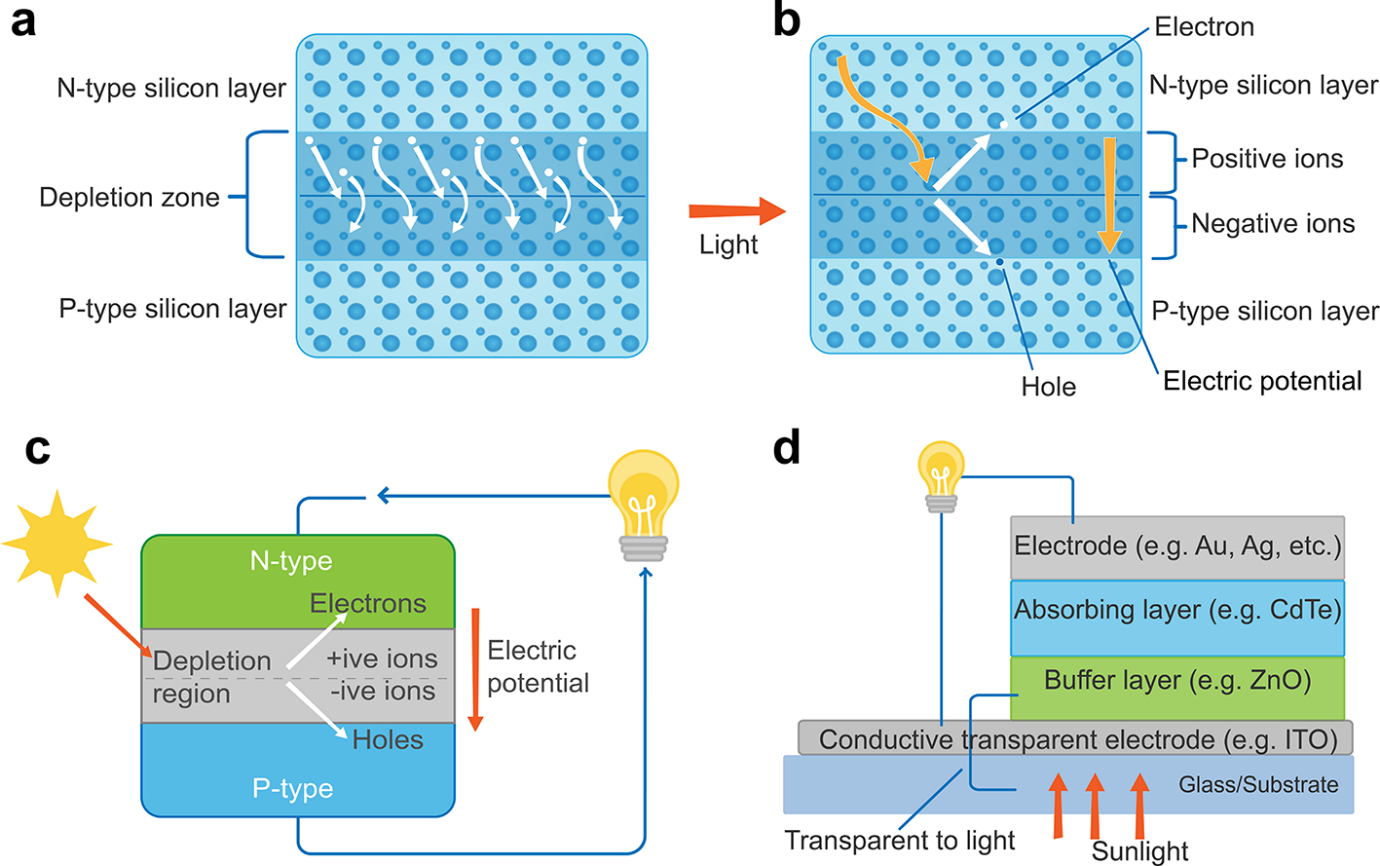
Power generating mechanisms and structure of photovoltaic systems. General mechanism of photovoltaic process in SCs (a) without sunlight illumination and (b) with sunlight illumination. Schematics of (c) first-generation and (d) second-generation SCs.

The construction of a SC is very similar to the concept of a p–n junction diode. An n-type and a p-type material are sandwiched together, and when they come close to each other, electrons from the n-type material diffuse into the holes of the p-type material, creating a depletion region in between. This depletion region acts as an active region that enables the photovoltaic mechanism. To better understand the SC mechanism, the basic structure of a SC is shown in Figure 4a and b. Since the majority of charge carriers (i.e., electrons in n-type and holes in p-type semiconductors) begin to diffuse once the two materials (p-type and n-type) are bonded, they leave exposed charges on the dopant atom sites, that are stuck in the crystal lattice and cannot move. In the n-type material, the centers of positive ions are released, while in the p-type material, the centers of negative ions are released. N-type materials have clusters of positively charged ions, and p-type materials contain clusters of negatively charged ions; an electric field E is generated between these two types of ions. Since free carriers are rapidly swept out of this area by the electric field, it is referred to as the “depletion region.″ The junction receives enough energy from the photons of light to generate multiple electron–hole pairs. The sunlight disturbs the junction’s thermal balance, exciting charges to create hole-pairs. Electrodes collect both positive and negative charges and transfer them to an external load.^138^

**Figure 5:**
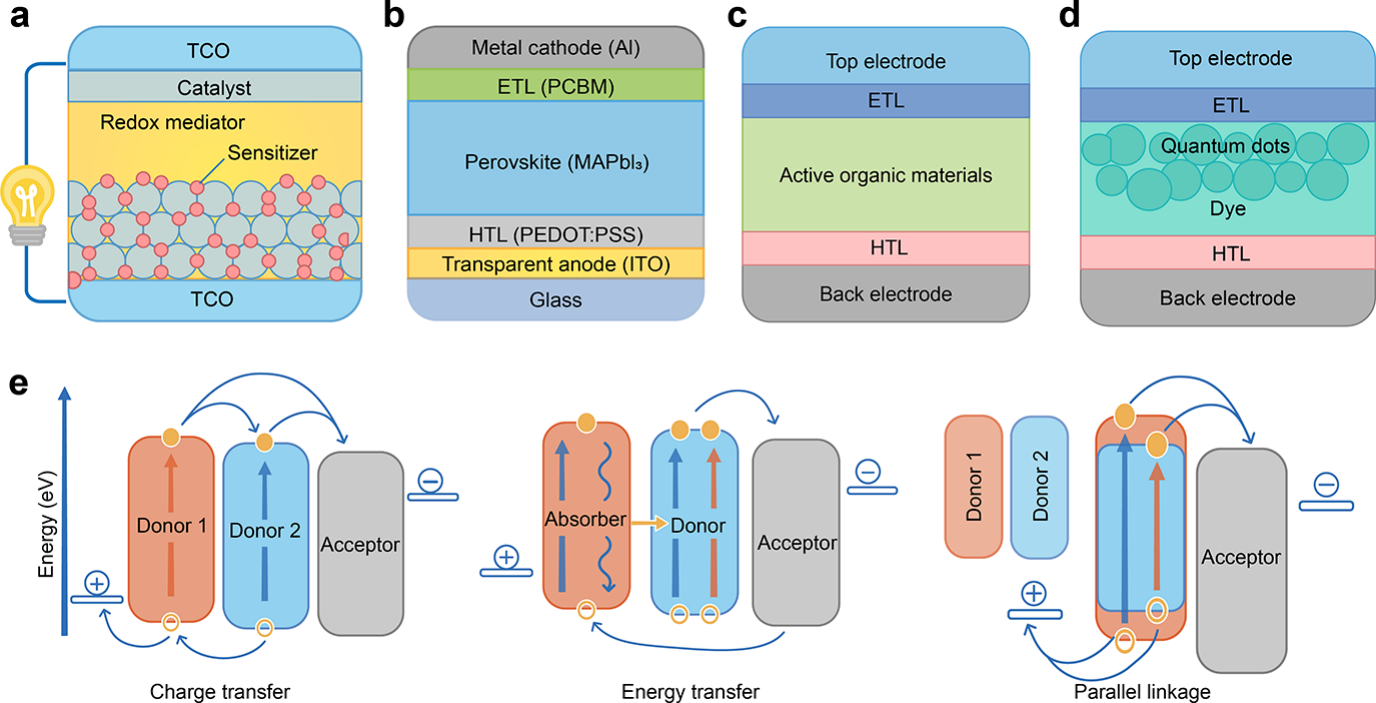
Schematics of photovoltaic technologies in third-generation SCs. (a) Dye sensitized solar cell (DSSC), (b) perovskite solar cell (PSC), (c) organic solar cell (OSC), and (d) quantum dot solar cell (QDSC). (e) The energy transfer mechanism of the photovoltaic process in third-generation OSCs.

Second- and third-generation SCs consist of several layers of light absorption materials that are up to 300 times thinner than Si-based SCs.^139^ Such SCs devices consist of n-type and p-type materials, also known as acceptor and donor materials, which form heterojunctions. Regardless of the structure or the material types, the essential function of each generation of SCs is the same, generating electricity directly from solar energy. In the third-generation of SCs, typically for the photovoltaic mechanism, there are 4 steps involved: light absorption, electron injection, transportation of carriers, and collection of current.^140^ In the photovoltaic process of third-generation SCs, especially organic solar cells (OSCs), photons (energy packets) from the sun are absorbed by the active layer (also known as an absorber layer), which consist of donor and acceptor semiconducting materials. Upon photon absorption, excitons (bounded electron–hole pairs) are formed, with the electrons dissociating at the lowest unoccupied molecular orbital (LUMO) level of acceptor and the holes are at the highest occupied molecular orbital (HOMO) level of donor. These dissociated electrons and holes are driven by the built-in electric field and then transported to the negative and positive electrodes, respectively. Concurrently, these dissociated electrons and holes are trapped in the charge carrier transport layers, the electron transport layer (ETL) and the hole transport layer (HTL).^141−143^ The electrons are collected by the external electrodes and recombine with the unoccupied holes after passing through the load, as illustrated in Figure 5e. The distinctive chemical and physical characteristics associated with each layer are outlined in the Layers and Materials for SCs section, along with the impact in performance of the SC device.

### Generation and Types of SCs

With an ever-increasing demand for energy, research, and development (R&D) on SCs has been extensive in recent years. To achieve the highest possible efficiency in SCs, researchers have been investigating both materials and modern fabrication technologies. Based on their development stages, materials and working processes, SC technologies may present in three separate generations:^144^ (i) first-generation, (ii) second-generation and (iii) third-generation.

### First-Generation SCs

First-generation SCs are a well-developed technology in the field of photovoltaics. These systems are widely used for commercial purposes. Figure 4c illustrates the general structure of first-generation SCs, which consists of a sandwich-type structure with n-type and p-type semiconducting materials. First-generation SCs are typically based on crystalline semiconductor films such as silicon (Si) and gallium arsenide (GaAs). GaAs, with a bandgap of 1.43 eV, is excellent for single-junction photovoltaic applications. In addition, GaAs have high absorption capabilities, allowing for high absorption spectra even with cells only a few micrometers thick. In contrast, Si-based cells require thicker layers to achieve appropriate absorption. Furthermore, GaAs-based SCs have a lower temperature coefficient, resulting in minimal temperature dependence.^145^ Thermal laminations have recently been used to develop transparent polyhedral oligomeric silsesquioxanes (POSS) polyamide film sealed flexible triple junction GaAs thin film SC with an outstanding power conversion efficiency (PCE) of 28%.^146^ While GaAs is one of the oldest materials used in first-generation SCs with higher PCE, Si has dominated the commercial market, capturing 90% of the market share, due to several advantages. These advantages include its abundance in the earth’s crust,^147^ and its nontoxic nature that prevents contaminations and enhances durability. Single crystal silicon SCs have received significant attention from scientists for the past half-century and were initially reported by Bell Laboratories in 1954.^148^ Since then, the SCs market has been greatly influenced by these advancements and they have played an important role in energy production in domestic and commercial sectors. Silicon-based SCs have had significant progress, reaching a PCE greater than 5% in 1957.^149^ Within three years, by 1960 the efficiency increased to 14%.^150^ In 1973, NASA launched a self-powered Skylab using silicon-based SCs.^151^ During that era, some other materials were also introduced for SC applications, such as AlGaAs-based SC in 1970. In 1995, a monolithic AlGaAs/Si-based SC was produced with a high conversion efficiency of up to 20.6% using 4 terminal configurations.^152^ The maximum theoretical efficiency for silicon-based SCs is limited to 34%, while the greatest achieved efficiency to date is 24.7%.^153,154^ However, despite the expansion of the silicon-based SCs business in recent years, it has been hindered by high costs.^155^ The expensive recovery and refining of Si, as well as its preparation into wafers, significantly contributes to the high price. The production process of Si is also complex and requires extremely high temperatures. Despite technological advancements, there have been only minor cost reductions in Si technology.^156^ Additionally, Si-based SCs require thick materials as they are indirect bandgap semiconductors. Therefore, for wearable electronics applications, it is more favorable to use low-cost, direct bandgap semiconductors that require less complex manufacturing technologies.^157^ Furthermore, first-generation SCs are not suitable for wearable electronics applications due to their larger size and the need for a fixed supporting substrate, such as glass.

### Second-Generation SCs

Second-generation SCs, including thin film technologies, are also known as thin film SCs. The primary objective of second-generation SCs was to reduce their cost and size, which were the main disadvantages of first-generation SCs. Figure 4d depicts the fundamental configuration of second-generation thin film SCs, which consists of four layers: the top electrode (TE) or conductive transport layer, an active layer (also known as the absorbing layer), buffer layers (e.g., ZnO) and counter electrode (CE) layers. The foundation of the second-generation SCs was achieved through the utilization of thin-film materials such as amorphous silicon (a-Si), cadmium telluride (CdTe), copper indium gallium selenide (CIGS) and some other materials with a direct bandgap, including copper sulfide (Cu_2_S) and cadmium sulfide (CdS). The use of these not only reduced the volume of materials but also the overall size of the device. Several manufacturers claimed an efficiency of ∼10% for Cu_2_S/CdS cells developed using the cleveite method.^154,158^ CdTe-based thin film SCs were introduced in the early 1970s, and to this day, it remains the sole thin film technology among the world’s top ten SCs manufacturers.^159,160^ CdTe is very strong with extremely good chemical stability, making it suitable for various scalable deposition techniques. According to the Schockley-Queisser limit, CdTe has an optimal band gap of 1.5 eV, allowing it to achieve ∼32% efficiency with an open circuit voltage (*V*_oc_) of more than 1 V and a short circuit current density (*J*_sc_) of more than 30 mA cm^–2^.^159,161^ Second-generation SCs are comparatively cost-effective as they are thin and lightweight. Additionally, they have excellent light-absorbing characteristics and can achieve a high PCE of around 22.6%.^162^ However, second-generation SCs are not as long-lasting as the first-generation since they degrade quickly in outdoor conditions.^163^ While the performance of second-generation SCs is continuously improving with lower production costs, the materials needed to produce them are rare and less accessible than those of the first-generation.

### Third-Generation SCs

The third-generation of SCs was developed almost after three decades of the first-generation and second-generation to address the limitations of second-generation technologies and aimed to introduce innovative materials using modern processes for building flexible SCs.^141^ Dye-sensitized SCs (DSSCs), perovskite SCs (PSCs), OSCs, and quantum-dot SCs (QDSCs) are among the most common photovoltaic technologies used in third-generation SCs.^164^

### Dye-Sensitized SCs

Dye-sensitized SCs, also known as DSSCs, are one of the prominent third-generation SC technologies. DSSCs typically have a sandwich-type structure, consisting of two electrodes stacked one on top of the other with an electrolyte (containing a suitable redox pair with an acceptable solvent) in between them (Figure 5a). They are capable of generating electricity in a wide range of lighting conditions, both indoors and outdoors, allowing users to convert both artificial light and sunlight into electrical energy.^165^ In recent years, DSSCs have emerged as potential competitors to Si-based SCs due to their easy and solution-based fabrication process and their cost efficiency. The history of DSSCs began in 1960 when it was discovered that illuminated organic dyes could be used as an electrochemical system to generate electricity.^166,167^ In 1972, scientists successfully developed the chlorophyll-sensitized zinc oxide (ZnO) electrode for electricity generation by injecting excited dye molecules into a semiconducting material.^166,168^ DSSCs have recently emerged as a promising technology due to their potential for achieving high PCE combined with low production costs and a straightforward fabrication process.^169^ Additionally, DSSCs are compatible with flexible and wearable substrates, making them highly versatile in various applications. For example, a textile-based DSSC has been recently reported,^170^ achieving an efficiency of up to 1.8% under air mass 1.5 global (AM 1.5 G) solar spectrum conditions. The global AM 1.5 G spectrum is designed for flat-plate solar units and represents the average yearly solar irradiation at midlatitudes, accounting for 1.5 times the typical surface atmospheric depth. while maintaining photoelectric output stability for 7 weeks. Significant progress has been made in developing environmentally friendly dye materials, including the use of natural dye collected from harda fruits resulting in a fabric DSSC that achieved a notable PCE of 3.52%.^150^

In fact, DSSCs are gaining popularity as their performance continues to improve, with a recent report of a 13% PCE.^164^ Additionally, the development method for DSSCs is simple and cost-effective. However, there are still significant challenges associated with DSSCss, including the use of liquid electrolytes, which pose durability and stability issues, especially with changing temperatures. Moreover, the electrolyte can freeze at a low temperature, directly affecting the electrical performance. Therefore, there is a pressing need to address these issues and to extend the lifespan of DSSCs to make them a competitive device in the commercial market.

### Perovskite SCs

Perovskites are a class of compounds that can effectively coat other surfaces and absorb significant amounts of sunlight. The term ‘perovskite’ is derived from the nickname given to its crystalline structure. PSCs have shown promise for high-performance and low-cost SCs, as depicted in the general schematic shown in Figure 5b. The chemical formula for perovskites can be written as ABX_3_, in which the letters “A” and “B” represent cations, and the letter “X” represents ananion that binds to both cations.^171^ PSCs have undergone tremendous development in recent years, achieving a substantial increase in efficiency.^172^ The fabrication process of PSCs is often performed at a low temperature, making them an excellent option for the roll-to-roll process and a suitable choice for flexible and plastic-based SCs.

The efficiency of PSCs has increased significantly from approximately 3% in 2009 to more than 25% in recent years.^173,174^ Recently, the pseudohalide anion format (HCOO^–^) was employed to reduce anion-vacancy defects at grain boundaries and surfaces of the perovskite layer, as well as to enhance the crystalline structure of the layer. The developed PSC device demonstrated a higher PCE of 25.6% with long-term functional stability for up to 450 h.^175^ In other recent research, PSCs were developed and achieved a significantly high PCE of up to 25.8%, by incorporating an interlayer between a SnO_2_ electron transport layer (ETL) and a halide perovskite layer. The inclusion of the interlayer improved charge collection and transportation from the perovskite layer, enhancing the overall device performance. Furthermore, the PSCs showed excellent stability for approximately 500 h and achieved 90% consistency in the performance.^176^

It is worth mentioning that although PSCs have achieved comparatively higher PCE, there are several challenges associated with these SCs that need to be addressed before successful commercialization. These challenges include device hysteresis, toxicity and the stability of perovskite’s materials. Additionally, the use of hazardous lead in perovskites raises environmental concerns. Further research in encapsulation is essential to mitigate any potential toxicity and hazardousness.

### Organic SCs

Organic SCs (OSCs) are another family of third-generation SCs in which the absorbing layer is composed of organic semiconductors, primarily polymers (e.g., poly(3-hexylthiophene-2,5-diyl) (P_3_HT), poly [[4,8-bis[(2-ethylhexyl)oxy]benzo[1,2-b:4,5-b′]dithiophene-2,6-diyl][3-fluoro-2-[(2-ethylhexyl)carbonyl]thieno[3,4-*b*]thiophenediyl]] (PTB_7_), etc.) or small molecules (e.g., dithienobenzodithiophene (DTBDT), benzodithiophene (BDT) etc.). A general schematic of an OSC is given in Figure 5c, consisting of several functional materials such as charge career transport layers, doner and acceptor layers etc. OSCs were initially developed in the 1950s but were not widely adopted due to their lower PCE.^177^ OSCs have gained significant traction due to their exceptional flexibility and compatibility with wearable devices, making them increasingly popular. Unlike traditional SCs that rely on crystalline semiconductor materials, OSCs utilize organic polymers or small-molecule materials, presenting a more cost-effective alternative for capturing solar energy. Although OSCs typically exhibit lower power conversion efficiency (PCE) compared to silicon-based SCs (ranging from 15% to 40% PCE), their significantly lower manufacturing cost, which is roughly one-tenth of that of silicon-based SCs, offers substantial advantages in terms of affordability and accessibility.^178^ It has been demonstrated that an OSC with a PCE of 5% can be achieved by employing the P3HT:PCBM as an active layer in a typical configuration.^179^ An inverted structure OSC using the active material P_3_HT:PCBM has been investigated to achieve a high PCE of 9.2%.^180^ Furthermore, an OSC based on P_3_HT and a fullerene derivative, indene-C70-bisadduct (IC_70_BA) acceptor material, achieved a PCE of 7.4% by utilizing a high boiling point solvent additive.^181^ Recently, OSCs have been reported with high PCE of up to 15.8% and excellent stability, maintaining over 86.6% of their initial performance after 1574 h in air under dark conditions at room temperature. Additionally, the impact of temperature was investigated by storing the fabricated OSCs for 172 h at a temperature of 85 °C, and a 92.4% similarity in performance was observed.^182^

Although OSCs are gaining popularity, they are still not comparable to Si-based SCs due to certain limitations including the use of fullerene derivatives (e.g., PCBM), which abruptly affect the transport of electrons and the kinetics of the recombination process.^183,184^ Other challenges include low absorption in the visible range and poor precise control of energy levels. The PCE of OSCs still fall short of the commercial standard value of 15%.^185^ Additionally, the lifetime of OSCs currently reported is relatively low, which is below the commercial threshold.^186^ Further research is needed to improve the durability and efficiency of OSCs to establish their market viability.

### Quantum Dot Sensitized SCs

Quantum dot SCs (QDSCs) are an emerging class of third-generation SCs, Figure 5d. These SCs utilize quantum dots as the light-absorbing photoactive material.^187^ Quantum dots (QDs) are nanoscale semiconductors created by humans and are widely used in various applications, including light-emitting diodes (LEDs) and SCs, due to their unique optical and electrical properties.^188−190^ QDs are ideal for use in multijunction SCs because of their unique properties of having an adjustable bandgap and giving an advantage over bulky light-absorbing materials having fixed bandgaps.^191−193^ In 1998, QDSCs with a power conversion efficiency of less than 1% were published^194,195^ Later on, several research groups recognized the potential of QDs for SC applications. QDSCs were found to have the ability to surpass the theoretical efficiency limit of 31% for single-junction cells.^190^ A recent demonstration of QDSCs using Zn–Cu–In–S-Se (ZCISSe) QD-sensitized TiO_2_ film electrodes achieved a maximum PCE of 15.31%.^196^ Other recent research showed that perovskite quantum dots (PQDs) based on CsPbBrCl_2_:Sm_3_^+^ achieved a PCE of up to 22.52%.^197^ Despite the advantages offered by QDs over bulk materials, the practical application of QD-based SCs is limited due to certain limitations. For instance, SCs based on cadmium selenide QDs are very hazardous, and require robust polymer packaging. The addition of an extra packaging layer increases the manufacturing cost and can impact device performance, including light absorption and PCE.

### Structure of Textile-Based SCs

**Figure 6:**
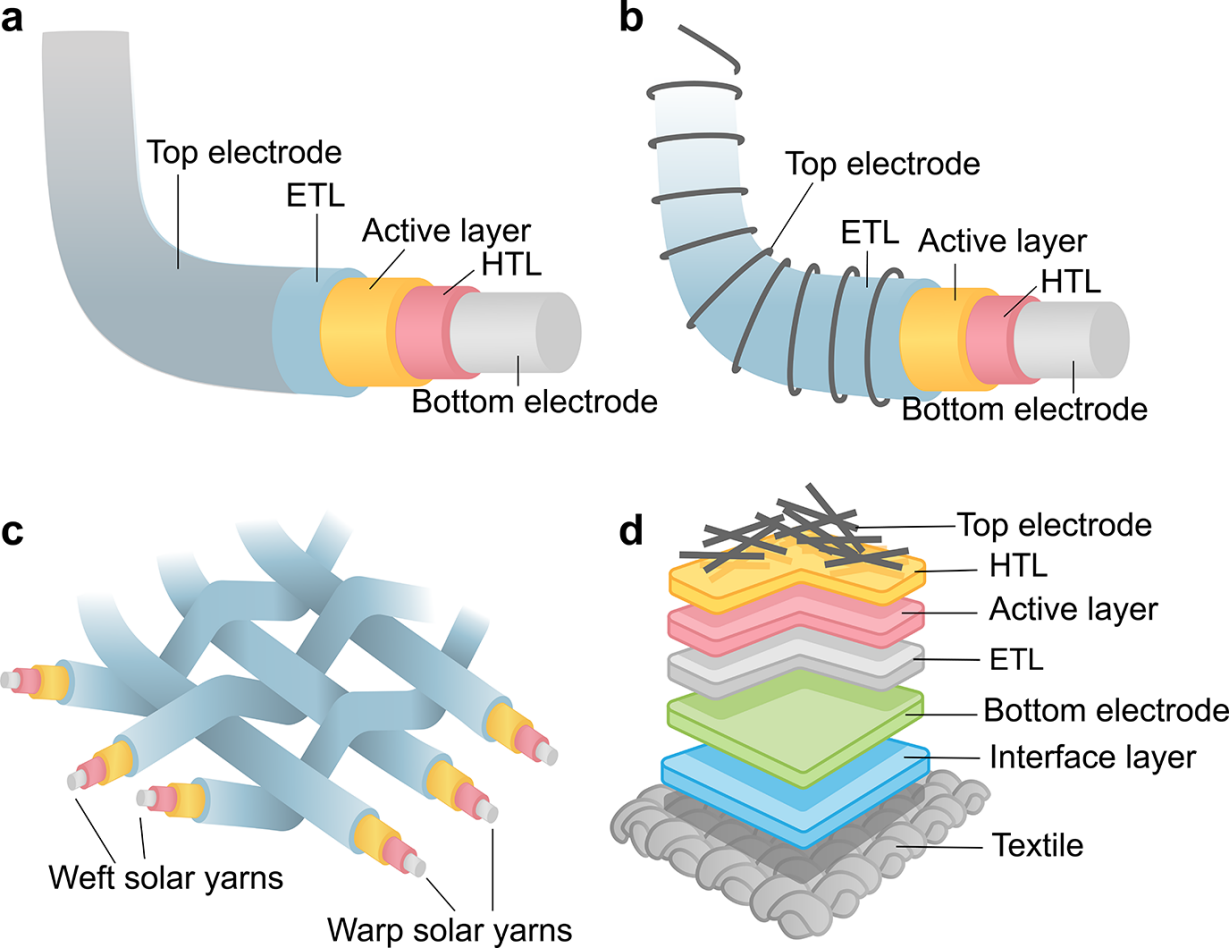
Schematics of photovoltaic textile architectures. 1D fiber-level SCs: (a) coaxial type and (b) twisting type; 2D textile-level SCs: (c) interlaced and (d) planar shape textile-based SCs.

Depending on the shape of SCs, textile-based SCs can be divided into two categories: 1D or fiber-shaped and 2D or planar-shaped SCs, as shown in Figure 6.

### Fiber Shape Textile-Based Solar Cells

Fiber-shaped textile-based SCs, also known as 1D SCs, are named as such due to their unique configuration. Early studies ignited interest in exploring the structural layout of 1D SCs. Depending on the configuration and spatial interaction between the electrode and functional layers, 1D or fiber-shaped SCs can be further categorized as coaxial, twisting, and interlaced designs.^198^

### Coaxial Type 1D SCs

The coaxial structure of 1D SCs is derived from planar devices, as all layers are grown on a single fiber substrate/electrode. A typical configuration of 1D devices is as follows: internal electrode (fiber shape)/ETL/absorption layer/HTL/external electrode, Figure 6a. The coaxial arrangement offers robust coupling of electrodes with functional layers such as HTL, resulting in superior charge collection and transportation due to the small carrier distance.^199^ This is why the coaxial structure is preferred in solid-state SCs like OSCs and PSCs.^200−202^ However, one drawback of this configuration is that it lacks transparency since the functional layers are completely covered by external electrodes, which affects light transmission. One possible solution is to use a thin layer to enhance transmittance, but this can increase resistance due to the reduced thickness of the conductive materials, directly impacting the device’s performance. Additionally, mechanical stability is curtailed for maintaining the longevity of the device.^203^ Moreover, the fabrication of tiny external electrodes and their subsequent connection to external connectors pose significant challenges in the manufacturing process^204,205^

### Twisting Type 1D SCs

As the name suggests, these 1D SCs have a twisted or spring-shaped structure. The device fabrication and configuration are the same as in coaxial-type SCs, but the exterior electrodes are twisted around in a spring form on HTL/ETL layers, giving additional room for light transmission, Figure 6b.^206,207^ These twisting-type SCs are significant to achieve high electrical performance due to the choice of sophisticated electrode materials and refined fabrication process. Furthermore, these fiber-based SCs are advantageous for DSSCs, due to their use of a liquid electrolyte, as the electrode makes full contact with the electrolyte and forms a better interface between them.^208,209^ Highly stretchable and flexible fiber-shaped OSCs, configured in a twisted design, have been demonstrated as a sustainable option for wearable applications.^210,211^ However, it is important to note that the shadow cast by the external electrode over the functional layer, as well as the longer charge carrier distance, can reduce the SCs performance of twisting-type SCs. In general, the thickness of the electrode fiber directly impacts the performance of twisting-type SCs. Furthermore, these twisted-type SCs tend to be less mechanically resilient compared to the coaxial type.

### Interlaced/Interwoven Type SCs

When it comes to the construction of large-scale textile-based SCs, the interwoven form of SCs is the most practical configuration.^212^ In an interlaced type of SC (Figure 6c), SC yarns are of are woven together. While this configuration enables the development of large-area Textile-based SCs, the contact between two adjacent fibers is not tightly secured, which limits significant charge collection. Additionally, the development of a dense and interlaced structure textile-based SCs may exhibit more shadows, which might have an impact on the overall performance.^165,213,214^

### Planar Shape 2D Textile-Based SCs

There are typically two different ways of constructing planar-type Textile-based SCs, also known as 2D SCs (Figure 6d). The traditional methods involve creating a flexible SC and then incorporating it into fabrics using adhesive materials.^215^ These techniques are straightforward and may not require additional fabrication standards, such as modifying the surface, apart from those specific to the SC type. Textile-based SCs created using these methods tend to have higher performance since they are affixed to textiles using adhesive tape, rather than modifying the original structure of the SC.^202,216,217^ However, these Textile-based SCs have limitations, such as reducing the garment’s washability and limiting flexibility and wearability due to their rigidity. An alternative strategy is the direct development of an SC device on textiles. This approach has gained interest in the flexible and wearable solar energy research community. However, several factors, including the rough surface of textiles and the additional layers (e.g., encapsulation materials), reduce light transmission and have a negative impact on SC performance.^109,218^

## Key Performance Metrics

**Figure 7:**
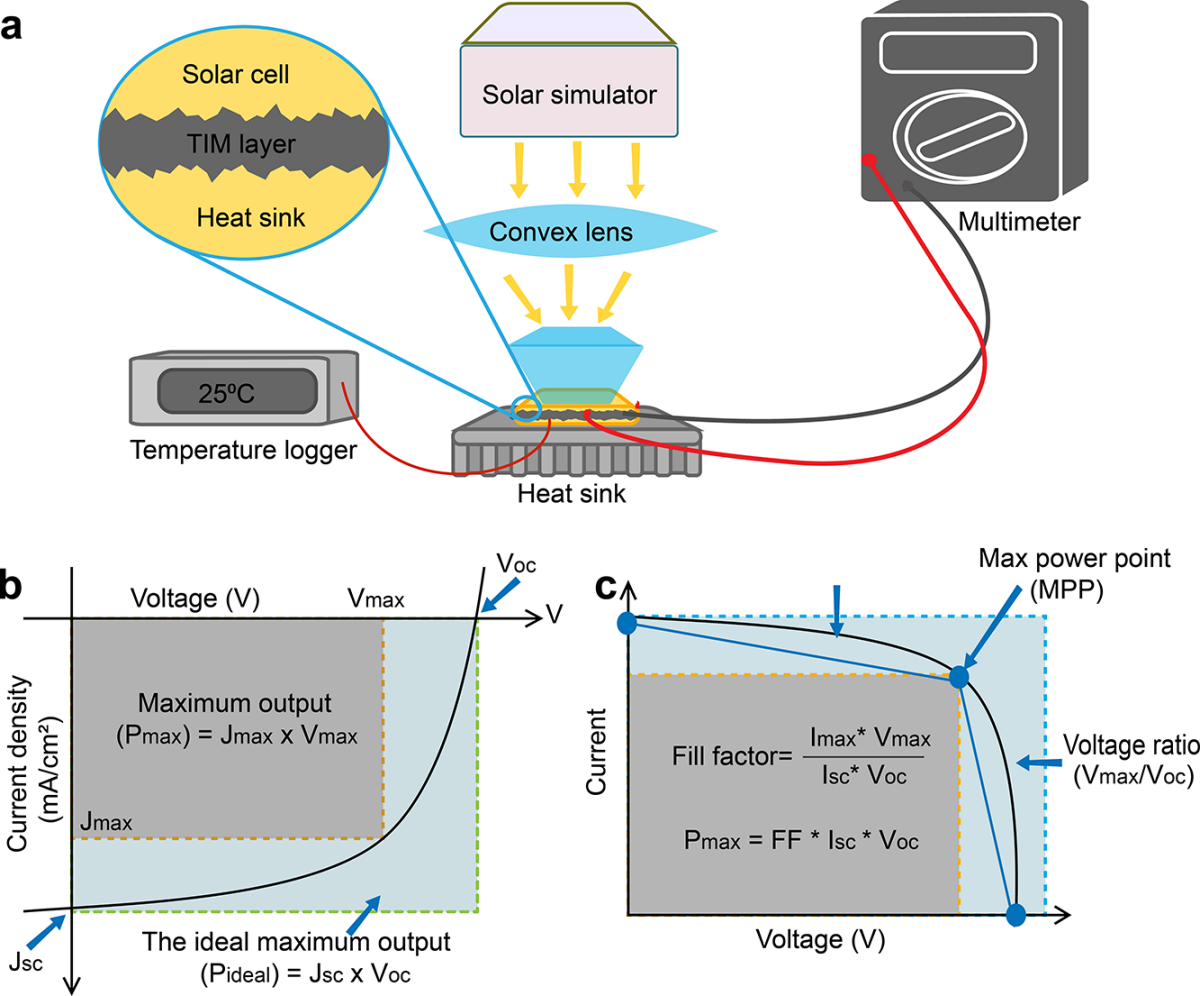
General photovoltaic system measurement setup and performance metrics. (a) Measurement setup for a SC characterization; graphical representation of (b) maximum power output and (c) the fill factor.

Every device has various essential aspects and qualities that determine its performance. SCs have fundamental specifications that must meet certain criteria to be considered sufficient for use. Figure 7a illustrates a general measurement setup, which includes a solar simulator along with electrical output measurement tools. The short-circuit current density (*J*_sc_) vs *V*_oc_ curve, along with other performance metrics of a SC, are shown in Figure 7b and c. These basic performance metrics of a SC include power conversion efficiency (PCE), fill factor (FF), maximum power (*P*_max_), ideal power (*P*_ideal_), open circuit voltage (*V*_oc_) and the short circuit current density (*J*_sc_).

### Power Conversion Efficiency

Power conversion efficiency (PCE) is one of the key and essential metrics that define and compare SC performance with other referenced SCs. PCE is defined as the ratio of generated maximum output (*P*_max_) to the input energy from the sunlight (*P*_in_) and it can be mathematically expressed as
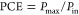
1In an ideal scenario, sunlight under AM 1.5 G conditions can provide *P*_in_ at the rate of *E* = 100 mW/cm^2^, then further simplifying the mathematical expression for PCE can be written as PCE = *P*_max_/*E* or can be calculated using FF (described in Layer-by-Layer Fabrication section) as the following equation:
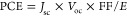
2SCs can be made more efficient using various approaches. One strategy to increase spectral efficiency is by employing a variety of semiconductor materials. Multijunction or heterojunction devices can achieve greater spectral efficiency by collecting diverse regions of the solar spectrum. The utilization of photons with energies exceeding the energy band gap of the semiconductors improves the performance of these photons releasing charge carriers with energies above the Fermi level. A multijunction device consists of separate single-junction cells stacked in order of decreasing bandgaps. The top cell absorbs photons with high energies and transmits the remaining photons to cells with smaller band gaps for absorption. Recent studies have demonstrated that by sequentially merging 36 SC junctions, an ideal PCE of 72% can be achieved.^219^ However, the development of these multijunction SCs is primarily hindered by their technical complexity and higher cost. It is worth noting that the PCE values that have been reported to date for textile-based SCs are relatively lower. For example, recently a textile-based DSSC with ∼3.86% PCE has been reported, utilizing carbon fabric/polypyrene as the reference electrode.^220^

Despite the considerable time that has passed since the development of single-junction SCs, the practical PCE of SCs remains around 27%.^221^ To achieve higher efficiencies, stacks of two or more absorber layers (multijunction cells) can be employed, enabling PCEs of more than 30%.^222,223^ Tandem SCs, which consist of two different types of SCs, are a rapidly advancing area of research. Tandem SCs have the potential to increase the PCE of SCs to more than 45%.^224^ In addition, the search for efficient materials and composites with tuned bandgaps (e.g., quantum dots) is an intensive area of exploration, which could lead to the utilization of the entire solar spectrum and further improve the PCE of SCs.

### Fill Factor

The fill factor (FF) is another essential performance metric that indicates how close a SC to an ideal device. It is a critical parameter influencing the PCE of SCs. The FF is the ratio of maximum power from the device (*P*_max_) to the ideal power (*P*_ideal_) and can be calculated either as,
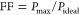
3or
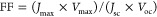
4The maximum FF reported for silicon-based SC is about 80%.^225^ Recently, 1 cm^2^ PSCs have been reported attaining a FF up to 85.3%.^226^ In the developed PSC device, a nitrogen-doped titanium oxide (TiOxNy) electron transport layer was used to construct the apparatus to facilitate the charge movement between the perovskite absorber and the electrodes. The fill factor can be improved by selecting materials with a lower resistance, as a higher resistance leads to an increased voltage drop. Additionally, having an optimum band gap and high absorption coefficient are other factors that contribute to improving the FF.

### Power Maximum and Power Ideal

The power of a device is a primary concern for consumers and is typically specified in the datasheet of the electrical devices. The maximum output power (*P*_max_) is an important parameter that helps determine the quality of a SC. In Figure 7b, the yellow region represents *P*_max_ which is the product of *J*_max_ and *V*_max_ under standardized sunlight energy conditions (AM 1.5 G) and is measured in mW/cm^2^ units:
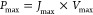
5For every device, ideal outcomes are anticipated but they are often impractical to achieve. These ideal outcomes serve as benchmarks to assess how close the actual results are and help to evaluate other metrics. In the case of SCs, there is a metric called the ideal power (*P*_ideal_), which is determined under ideal conditions. Under these conditions, the output current is considered maximum while the voltage drop is assumed to be zero (short circuit), and vice versa for measuring the output voltage (open circuit). In simple words, *P*_ideal_ is the product of *J*_sc_ and *V*_oc_, which will be discussed in the following section. Mathematically, *P*_ideal_ can be represented as
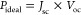
6

### Current Density

Current measurement is a crucial output in any energy-generating device, as it helps describe other parameters such as power. In general, current refers to the flow of charged particles, such as electrons or ions, and its SI unit is the ampere (A). In a SC the amount of current generated depends on the amount of active area exposed to solar energy. Therefore, current is typically measured per unit area and is referred to as the current density (*J*). One commonly used term in creating the IV curve is the short circuit current density, denoted as *J*_sc_. It represents the maximum current when there is no voltage applied, as shown in Figure 7b and c. *J*_sc_ is measured by short-circuiting the electrodes of the SCs using a digital multimeter. Another important metric associated with current measurement is the maximum current density (*J*_max_). *J*_max_ represents the maximum output current that a SC can generate under specific load conditions. For silicon-based SCs under AM 1.5, the theoretical maximum current density can reach 46 mA cm^–2^.^227^ Lab-based devices have recorded short-circuit currents over 42 mA cm^–2^, while commercial SCs typically range between 28 and 35 mA cm^–2^.^228,229^

### Voltages

Output voltage is another important metric in the characterization of SCs. Voltages are measured under two conditions: with no load and under load. The *V*_oc_ represents the maximal voltage that a SC device can produce when no current is flowing to an external load or circuit (under no load condition), as shown in Figure 7b and c. In the no-load condition, the wires of the digital multimeter are directly connected to the anode and cathode of the SC devices, creating an open circuit system for measurement. *V*_oc_ corresponds to the forward bias voltage at which the dark current density compensates for the photocurrent density. Output voltage can also be measured under a specific load condition, and the highest value obtained is referred as *V*_max_. This measurement helps in calculating the maximum power *P*_max_ of the SC. Recently, a wide bandgap OSC with a *V*_oc_ of 1.4 V was reported, which represents the highest voltage reported for OSCs to date.^230^

## Layers and Materials for SCs

### Electrodes

Electrodes play a fundamental role in electronic devices as they are responsible for supplying or drawing electric charges to or from the device. In the case of SCs, the choice of electrodes directly impacts the efficiency and other performance metrics. Therefore, selecting suitable electrodes is crucial for optimizing the performance of an electronic device. In general, SCs consist of two electrodes: the bottom electrode and the top electrode. These electrodes can be made of various conductive materials, including metals, carbon-based components, conductive oxides, or conductive polymers. Table 1 summarizes commonly used electrodes for SC applications highlighting their advantages and limitations.

**Table 1:** Performance and Stability of Some Common Electrodes in SCs

Electrode Material	Configuration	Efficiency	Stability/Remarks	Ref
Ag	Ag/MoO_3_/PM6:Y6/ZnO/PH1000/Ag-Grid	10.87%	The photolithographic based Ag grid demonstrated high efficiency at 400 μm thickness (up to 97.6%)	(375)
Ag	FTO/Cu:NiO_*x*_/MAPbI_3_/PCBM/Ag	15.87%	Corrosive behavior of Ag electrode causes instability in PSCs	(376)
Ag thin film	TeO_2_/Ag/BCP/PCBM/Pervoskite/NiOx/ITO/Glass	17.36%	TeO_2_ with Ag is a good choice for developing bifacial SCs	(377)
AgNWs	Glass/FTO/bl-TiO2/MAPbI3/spiro-OMe-TAD/AgNWs	10.64%	AgNWs are found to be more stable in comparison to other metallic films	(239)
Au	DSSC	2.3%	Gold leaf based counter electrodes were found to be excellent for DSSC due to their porous surface	(378)
Au	Glass/FTO/SnO_2_/TiO_2_/Pervoskite/Spiro-OMe-TAD/Au	25.8%	In metal based electrodes Au is found to be the most stable	(176)
Au thin film	MoO_3_/Au/MoO_3_/PTB7:PC71BM/Al	6%	50% increase in PCE occurred in comparison to referenced device	(379)
Ni	Glass/FTO/TiO_2_/Al_2_O_3_/HP/piro-OMe-TAD/PEDOT:PSS/TCA/PET/Ni/PET	15.5%	PCE drops with the illumination of Ni (bottom electrode)	(279)
Cu	ITO/PTAA/MAPbI3/Cu	20.7%	Stays stable while maintaining 98% of original PCE after keeping for 816 h in ambient conditions	(274)
Al	ITO/PEDOT:PSS/P3HT:PCBM/LiF/Al	4.6%	The stability was improved with the insertion of an LiF layer in between the Al and active layer	(380)
Al	Glass/ITO/PEDOT:PSS/MAPbI3/PCBM/Al	16.1%	Due to oxidation the stability of Al is considerably low	(272)
CNTs	SWCNTs-Glass/FTO/C-TiO_2_/TiO_2_:Al_2_O_3_/MAPbI3/SWCNT-C	14.7%	Found to be more stable than metallic based electrodes	(381)
GR	PEN/GR/MoO3/PEDOT:PSS/MAPbI3/C60/BCP/LiF/Al	16.8%	Found to be comparatively more stable	(382)
GR	GR/PCBM:GQDs/MAPbI3/PTAA/Au	16.4%	Highly stable	(382)
GR with AgNWs	Gr:AgNWs/PH1000/PEDOT:PSS/Active layer/PDINO/Al	13.4%	Retained 84.6% PCE after being bent 1000 times.	(383)
PANI+Au		6.71%	PCE of the DSSCs with PANI/Au composite electrode was found to be more efficient compared to Pt based DSSC (PCE 6.18%)	(384)
ITO	Glass/ITO/PEDOT:PSS/Perovskite/PCBM/Ag	15.6%	ITO is a considerably stable transparent conductive oxide	(385)
polypyrrole (PPy), PEDOT		PEDOT (1.35%), PPy (0.41%), PT (0.09%)	PEDOT PCE was found to be comparable to that of a sputtered Au based reference device.	(386)

### Metal-Based Electrodes

**Figure 8:**
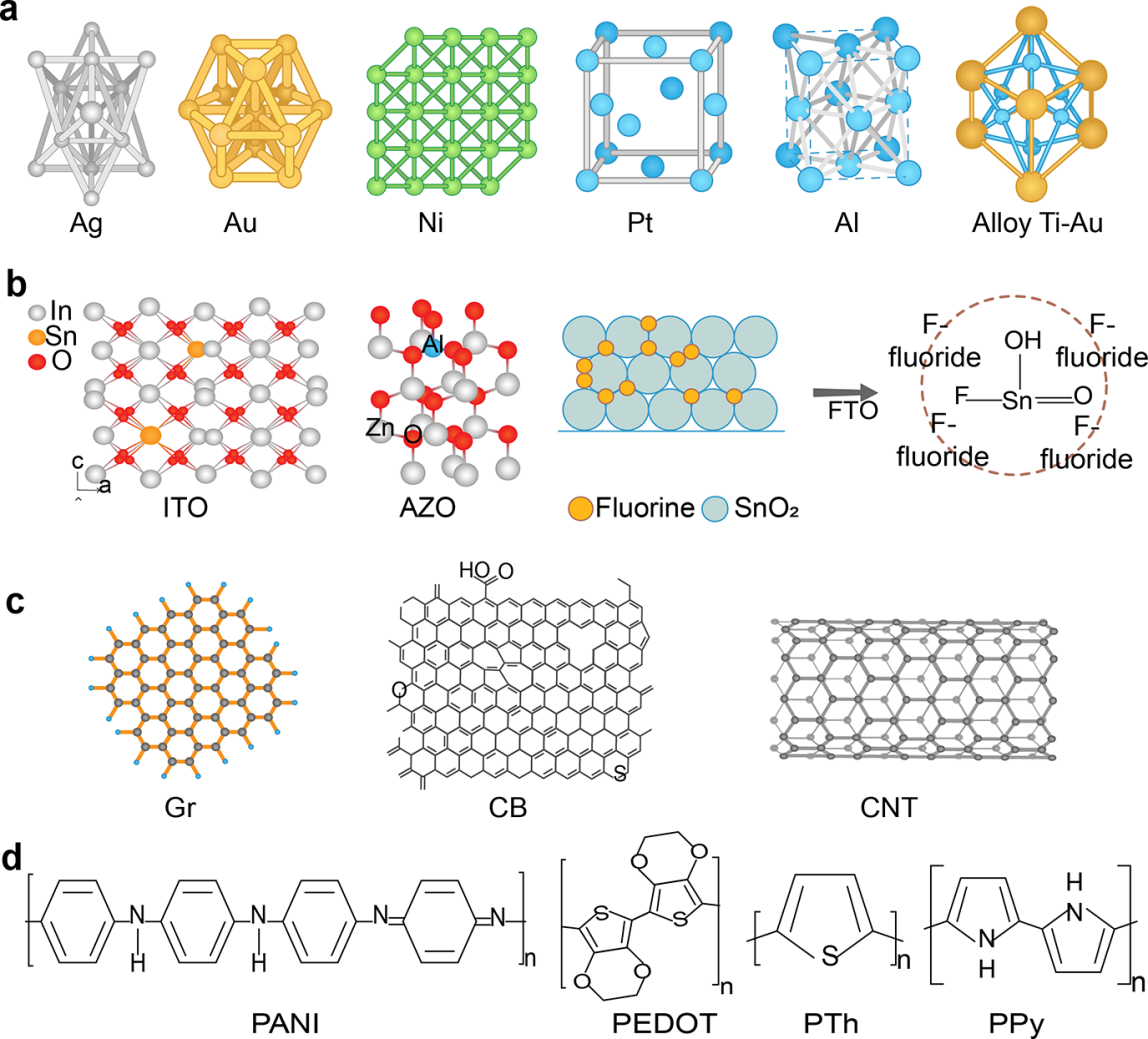
Chemical structures of common electrode materials employed in photovoltaic systems. (a) Metals, (b) transparent conductive oxides (TCOs), (c) carbon-based, and (d) conductive polymers.

Metals have been widely utilized as electrodes and conductive materials for many centuries. In the context of SCs metal electrodes are commonly employed for both the bottom and top layers. Some frequently used metal electrodes include platinum (Pt), aluminium (Al), gold (Au), and silver (Ag).^231^ The selection of a specific metal is typically based on factors such as its work function, resistance, and compatibility with other layers in the SC, such as the hole transport layer. The resistance, also known as contact resistance, between the transport layer and the electrode is crucial in determining the efficiency of a SC. Additionally, the transparency of the top electrode plays a significant role in reducing reflection.^232^ Therefore, thin metal-based layers, patterns or nanowires are being utilized as top electrodes.^233−235^ The crystal/chemical structure of commonly used metal-based electrodes is illustrated in Figure 8a.

### Silver

Silver (Ag) is a highly conductive metal with a work function of 4.3 eV. The work function refers to the minimum energy required for an electron to escape from the surface of a material. Ag has been commonly used as an electrode in various electronic devices. Due to its compatibility with different functional materials, it has been extensively used as an electrode in SCs for a long time. Ag exhibits good compatibility with the ETL layers in SCs, as well as being opaque in nature. It is often utilized as the bottom electrode. However, a thin layer of Ag and as well as silver nanowires (AgNWs) has been investigated as a top electrode material. Furthermore, Ag offers advantages such as its availability in various forms, including dispersions, pastes, and inks, which facilitate the deposition of Ag-based conductive layers for diverse applications, particularly in wearable and e-textile-based devices. While it has a suitable work function for some ETLs, such as zinc oxide (ZnO),^236^ it may degrade when used with certain other materials. For example, the interaction between Ag contacts and lead perovskite materials was investigated by depositing Ag directly onto the perovskite surface using thermal evaporation techniques. Instead of forming a uniform Ag layer, the deposited Ag formed particles on the perovskite layer, leading to the degradation of both the silver electrode layer and the perovskite layer.^237^ This highlights the challenges associated with using Ag in certain configurations. Additionally, AgNW electrodes are getting considerable interest due to their tunable optoelectronic and mechanical properties. However, the junction sites of AgNW electrodes often exhibit poor contact, resulting in increased sheet resistance and reduced mechanical properties.^238,239^ In 2012, Ag was employed in PSCs, while achieving a PCE of 10.9%.^240^ Ag has been employed in various generations of SCs, including first-generation silicon-based SCs,^241,242^ second-generation SCs,^243,244^ and third-generation SCs.^245−247^ In a recent study,^239^ silver ink has been spin-coated on a flexible PET substrate to fabricate PSCs with a PCE of 10.3%. Researchers have recently synthesized Ag nanowires and utilized them as top electrodes in different types of SCs.^248,249^ AgNWs have also been inkjet-printed as the top electrode of a SC, demonstrating a PCE of 2.7%.^250^ Additionally, the use of a Ag nanoparticle ink as the top electrode in a PSC, fabricated through spin coating techniques, resulted in a PCE of 10.3%.^251^ The literature on Ag as part of SCs indicates that Ag is one of the most common elements used in these devices,^237,252−254^ but it is important to address and enhance mechanical properties to maximize flexibility when considering wearable textile-based electronics.

### Gold

Gold (Au), a precious metal, has long been a commonly used and highly efficient electrode material in high-end devices due to its excellent conductivity.^255^ Au is predominantly utilized as a back electrode in SCs, but thin layers of Au have been investigated as a top electrode.^256−259^ In addition to its functionality as an electrode, Au has also been utilized as a buffer layer with other conductive materials to enhance the transmittance of the top electrode. For instance, the intercalation of ultrathin Au seed into V_2_O_5_/Au/Ag/V_2_O_5_ (VAuAgV) multilayers improves the optical and electrical characteristics of such structures. By reducing the thickness of the intermediate metallic layer, the optical transmittance can be increased by more than 15%.^260,261^ A recent study explored the use of an Au film between the emitter and base of a structure made of Si semiconductor diode structure to enhance the efficiency of SCs. The results showed that the voltage and current in the device incorporating the Au film were up to 10 times higher than in the reference device.^262^ In a comparative study of metal electrodes used in PSCs, it was reported that an Au electrode is the optimized choice for improving the efficiency and stability of the PSC’s.^263^ Recently, a 100 nm thick Au electrode was coated onto a glass substrate using thermal evaporation, resulting in a high efficiency of 25.2%.^263,264^ Additionally, Au has been utilized as the back/bottom electrode in PSCs. In a recent development, nanoporous gold back electrodes were formed using spin coating, leading to a PSC device with an efficiency of 7.99%.^265^ Furthermore, Au was employed as the top electrode in a tandem SC stacked on top of a Si-heterojunction device, achieving a high PCE of 28.3%.^258^

Au has also been employed in various e-textile and wearable applications. Recently, an Au textile-based electrode was utilized as the top electrode in an OSC using a physical lamination approach, resulting a PCE of 2.91%.^266^ Additionally, in another study, a yarn-based OSC with stainless steel wire as the primary electrode and Au as the secondary electrode achieved a PCE of 4.6%.^267^ While Au electrodes exhibit behavior similar to platinum, their use in the positive potential range is limited due to surface oxidation. It is evident that Au is a good conductor compared to other conductive materials; however, its high cost makes it commercially unfeasible for energy-harvesting devices, especially in textile and wearable electronics where cost efficiency is a key consideration.^268^

### Aluminum

Aluminum (Al) is considered one of the most suitable and cost-effective electrode materials used in SCs to date. Its work function is compatible with SCs and it has been utilized as an electrode in various types of SCs, including Si-based SCs,^269^ OSCs^270^ and PSCs.^271^ Recently, solution-processed PSCs with an Al electrode demonstrated a PCE of 16.1%, showing improved stability and reduced susceptibility to degradation compared to traditional PSCs.^272^ In recent research, the use of Al foil as a bottom electrode resulted in a PCE of up to 7.09%.^273^ Despite being less expensive and having an excellent work function, Al is prone to degradation in open air and water. It can oxidize and diffuse into the transport layers, altering their fundamental characteristics and potentially leading to issues such as short circuits and increased internal resistance.^231,274^

### Nickel

Nickel (Ni) is an abundant metal known for its low cost and high conductivity (14.3 MS/m^2^), which makes it the preferred material for electrodes and has been used since the early stages of SCs technology.^275,276^ Recently, a 1 μm thick electroplated Ni film was employed in a Si SC to enhance the Ohmic contact, resulting in a significant decrease in sheet resistance and higher FF of 81.2%.^277^ Ni electrode-based PSCs were investigated and compared to reference Au-based PSC devices, achieving a comparable PCE of 10.4% compared to 11.6% for the Au-based device.^278^ Furthermore, a Ni grid was evaluated as a semitransparent electrode with transparent adhesive materials for fabricating PSC devices, achieving a POCE of up to 15.5%, which is comparable to devices based on noble metal electrodes such as Au, and Ag.^279^ Ni has also been utilized in SCs as a composite material, such as nickel oxide (NiO) and nickel sulfide (NiS).^280,281^ A Ni acetylacetonate (Ni(acac)) precursor was used to fabricate NiO thin films in OSC device fabrication, resulting in a PCE of up to be 5.2% after heating the NiO layer to 400 °C and treating with oxygen plasma.^282^ Furthermore, Ni has been employed in textile-based SCs. For example, such as coating it as a counter electrode over cotton fabric in a textile-based DSSC resulting in a PCE of up to 3.83%.^283^ While Ni can serve as an alternative material to expensive metals at a significantly lower cost for SC fabrication,^276^ it does come with certain risks. Ni can cause allergies, heart and kidney diseases, lung damage, and nasal cancer, when in contact with the human body.^284^ These potential health risks may limit its applications in textile-based wearable energy harvesting.

### Platinum

Platinum (Pt) is known of being one of the most expensive and rare conductive materials, characterized by its high work function of ∼5.65.^285^ Pt is also biocompatible, making it an excellent choice for implantable biomedical devices.^286^ The use of Pt in SCs can be traced back to 1877, when it was employed as connections at the ends of a small cylinder of glassy selenium, marking the initial documentation of photoconductivity in selenium.^287^ Pt has been utilized in various types of SCs, including Si-based SCs,^288,289^ and DSSCs.^290^ In the development of photoelectrochemical SC, platinum islands ranging in size from 5 to 50 nm were deposited on n-type (n-Si) wafers. This configuration resulted in a maximum generated *V*_oc_ of 0.63 V, which is 8% higher than the *V*_oc_ produced by conventional p–n junction SCs.^291^ Pt has been recently utilized in a flexible DSSC, where the Pt electrode was printed on ITO-PEN through screen printing, resulting in a maximum PCE of 5.41%.^292^ In another application, a Pt/Ti bilayer was deposited using vacuum sputtering on ITO-PEN substrates to create a completely polymer-based DSSC, which demonstrated a PCE of 4.31%.^293^ Furthermore, Pt has been employed as a counter electrode in textile-based SC.^294^ While Pt is a favorable choice for electrodes due to its properties, it is important to note that Pt reserves are being depleted leading to an increase in device manufacturing costs. Consequently, Pt is not an optimal choice for applications where cost efficiency is a major concern such as SCs energy generation.

### Alloys

Alloys consist of two or more materials, including at least one metal, combined to enhance mechanical, electrical, and optical properties. In recent developments, an Ag–Al alloy was utilized as a cathode in PSC fabrication, resulting in a PCE of 11.76% and a higher *V*_oc_ compared to the standard device with ITO/PEDOT: PSS/CH3NH3PbI3/PCBM/Ag, as well as Al alone as a cathode.^295^ Furthermore, DSSC devices with alloyed (Ni–Pt) electrodes have been reported, achieving a PCE of 8.29% and 7.41% for DSSCs with Ni–Pt nanowire and nanosheet alloy electrodes, respectively.^296^ Alloys, with their ability to offer a wide range of mechanical and electrical properties, hold tremendous potential for the future of wearable applications.

### Transparent Conductive Oxides

Transparent conductive oxides (TCOs) are semiconductive materials with high energy bandgaps, offering excellent electrical properties and a high transmission capacity in the visible and near-infrared ranges. They have recently, garnered significant attention as crucial components in large-area electronics, including organic light-emitting diodes (OLEDs),^297^ liquid crystal displays (LCD),^298^ SCs,^299^ and as anti-reflective coatings.^300^ In the context of SCs, TCOs are employed as transparent electrodes to allow sunlight penetration along with charge flow but have the potential for counter electrodes as well.^301,302^ Additionally, TCOs are employed as buffer layers in SCs.^303^ They serve as alternatives to CdS in thin-film SCs like CuInS_2_, enabling the production of Cd-free SCs.^304^ Common TCOs include indium tin oxide (ITO),^272,305^ fluorine-doped tin oxide (FTO),^306^ and aluminium-doped zinc oxide (AZO),^299^ as shown in Figure 8b. Recently, various TCOs, including ITO, AZO, and FTO, with thicknesses ranging from 10 to 200 nm, have been investigated. Among them, ITO-based perovskite SCs achieved a maximum PCE of 10.06%, with *V*_oc_ = 0.84 V, *J*_sc_ = 18.92 mA/cm^2^, and FF = 60%. The performance of the other two TCO-based devices (AZO and FTO) was quite similar, with PCEs of 10.21% and 10.0%, respectively.^307^ Indium tin oxide (ITO) is widely used in SCs due to its high conductivity (>103 S/cm) and excellent transparency (>90%) in the visible range. It also has a higher work function compared to other TCOs.^308^ A PSC device with the configuration of ITO/Perovskite/Spiro-OMeTAD/Au attained a PCE of 13.5%.^309^ In another research work FTO was employed and an ETL-free PSC with the architecture FTO/Perovskite/Spiro-OMeTAD/Au was developed, reaching a PCE of up to 16.1%, which is comparable to that of a PSC device with a ZnO based ETL.^310^

Although TCOs, such as ITO, offer excellent performance potential, they are the most expensive part of SCs.^311^ For example, the cost of a TCO is approximately ten times higher than that of the perovskite layer. Additionally, TCOs, like FTO, are fragile and can cause degradation in PSCs when subjected to bending.^312^ Furthermore, their resistance increases with temperature, limiting the usability of SCs in high-temperature environments. It is important to explore alternative materials for transparent electrodes that can optimize both the mechanical and electrical characteristics. For example, research is being conducted on metal nanowires (NWs) such as Au and Ag NWs, as well as other conductive materials like PEDOT: PSS.^312,313^ These alternatives aim to address the limitations of TCOs and enhance the overall performance and durability of SCs.

### Carbon-Based Materials

Carbon-based materials are highly promising as electrode materials in SCs due to their excellent electrical conductivity, mechanical and electrical stability, and large surface area.^314−316^ These materials have been extensively studied in SCs research over the last two decades, particularly in Si-based SCs, where carbon nanotubes (CNTs) have been investigated as a means to reduce manufacturing costs.^317,318^ Furthermore, carbon-based materials have generated significant research interest for their application in emerging third-generation SCs technologies such as DSSCs and PSCs.^319−321^Figure 8c illustrates the crystal structures of some common carbon-based materials.

### Graphene

Graphene, a carbon allotrope consisting of a single layer of atoms arranged in a 2D honeycomb lattice nanostructure, has attracted significant interest since its demonstration in 2004 for various applications.^322−326^ Due to its excellent electrical conductivity, mechanical stability, and transparency graphene is well suited for use in heterojunction SCs.^327−330^ Its versatility allows for device applications, including as anodes, cathodes, and donor/acceptor layers.^331−334^ In recent research, an OSC device was developed using a solution processing method, where a single layer of graphene on a quartz substrate was employed as a transparent electrode. The device, with graphene as the transparent electrode, achieved a PCE of 0.4%, which is half the value attained by the ITO-based referenced OSC device.^335^ Another study found that applying a thin molybdenum oxide (MoO_3_) film to the surface of a 4-layer graphene stack achieved a PCE of 2.5%, which is comparable to the 3% achieved with the more expensive ITO layer.^336^ Recently, a semitransparent flexible OSC that acquired a PCE of 3% was investigated. Where the top electrode was formed by chemical vapor deposition (CVD) using graphene.^337^ Similarly a recent report highlighted the use of graphene as the top electrode in a PSC device, achieving an impressive PCE of up to 11.5% and demonstrating excellent flexibility.^338^ Graphene has also found applications in textile-based SCs. A metal-free DSSC utilizing carbon fabric coated with reduced graphene oxide (rGO) as the counter electrode has been developed, showcasing a noteworthy PCE of 2.52%.^339^ Furthermore, in another study, a highly conductive graphene-coated fabric was employed as a counter electrode in textile-based DSSCs, achieving a PCE of 6.93%.^340^ These graphene-based electrodes not only provide a cost-effective alternative but also contribute to reducing the manufacturing costs associated with the deposition of metal electrodes.^341^

### Carbon Nanotube

Carbon nanotubes (CNTs) are nanoscale hollow cylinders consisting of graphitic carbon atoms with a diameter much thinner (∼1 nm) than a human hair (10s of μm).^342^ Since their discovery in 1991, CNTs have captured worldwide interest for a variety of applications.^343^ Due to their unique photoelectric properties, CNTs have been extensively researched for their potential application in light-harvesting devices.^344^ CNTs have been employed in various types of SCs, including a Si-based SCs,^345^ OSCs,^346,347^ DSSCs,^348^ copper indium gallium diselenide (CIGS) thin film SC,^345^ and more. Recently, a PSC device with the configuration TiO2/perovskite/CuSCN/CNTs has achieved an impressive PCE of 17.58%. The developed PSC device has demonstrated an enhanced charge collection at the CuSCN/CNT layer contact.^349^ CNTs in combination with other conductive materials have demonstrated higher efficiency compared to those using metallic electrodes.^321^ For instance, a planar PSC with the device structure TiO_2_/MAPbI_3_/CNTs and copper(II) phthalocyanine (CuPc) as a carbon electrode modifier achieved a PCE of up to 18.8%, which was 30% higher than devices with a simple carbon electrode.^350^ Recently, a flexible OSC device with multilayer electrodes composed of CNTs and AgNWs reported a PCE of 2.21%.^351^ To maximize the efficiency of reflected light, a bifacial carbon-based PSCs (C-PSCs) with transparent CNT layers in the back electrode has been investigated achieving a PEC of up to 21.4% under natural sunlight conditions (20% of AM 1.5 G radiation) and 34.1% in artificial sun simulators (100% of AM 1.5 G irradiance). Along with that in a four-terminal (4-T) tandem configuration, where the bifacial device served as the top cell, combined with a CIGS bottom cell, a very high PCE of 27.1% was achieved.^352^ CNTs are gaining popularity in wearable and textile textile-based SCs as well due to their flexible nature. For example, yarn-based CdSe SCs directly utilize CNT yarns as the counter electrode and achieved a PCE of 2.9%.^353^ Additionally, CNT fibers have been utilized in the development of fiber DSSCs, resulted in a PCE of 2.94%.^354^

### Carbon Black

Carbon black (CB) is a term used to describe black materials produced through the partial combustion or carbonization of organic materials such as natural gas, oil, wood, and vegetables.^355^ CB materials are gaining popularity in SCs due to their affordability and ability to be deposited through solution processing. In Pt-free DSSCs, CB has been utilized as a catalyst for the reduction of triiodide on FTO glass substrates acting as counter electrodes, resulting in a PCE of 9.1% under light intensity (AM 1.5 G).^356^ In a research study, it was observed that the catalytic activity of CB increases as the particle size decreases. In this study CB particle sizes from 90 to 20 nm were employed as a counter electrode in DSSCs, with a particle size of 20 nm, resulted in a high PCE of 7.2%.^357^ In 2013, CB was investigated as a counter electrode in a PSC and a PCE of 6.6% was obtained.^358^ Low-temperature cured carbon electrodes were developed as substitutes for noble metals in HTL-free PSCs, attaining a PCE of 8.31%. This was further improved to 9% compared to the referenced device by utilizing a doctor-blading technique.^359^ Another approach involved the incorporation of a conductive silicone rubber layer loaded with carbon black and graphite on a viscose woven fabric to serve as a counter electrode with an intrinsic catalyst for textile-based DSSCs.^360^

### Conductive Polymer

Conductive polymers (CPs) are organic materials that possess metal-like properties, including electrical, optical, and magnetic capabilities, while also exhibiting fundamental polymer-like features such as being lightweight and possessing high stiffnesss and strength.^361^ Due to their cost effectiveness, low density, stretchability, and flexibility, conducting polymers have gained preference over other electrode materials.^362−364^ Researchers have extensively investigated the unique and interesting characteristics of CPs for the compatibility with various functional materials, development of smart sensors and systems, Organic LEDs, SCs, and a broad range of other electronic devices.^365,366^ Over the past three decades, several types of air-stable conducting polymers have been developed, with polyaniline (PANI), polypyrrole (PPy), and poly(3,4-ethylenedioxythiophene) (PEDOT) being the most commonly used CPs.^367,368^ The crystal structures of some common CPs are shown in Figure 8d.

A recent study investigated the use of PEDOT as a counter electrode with varying thicknesses. The researchers discovered that DSSC devices with a thin PEDOT layer (33 nm) achieved a PCE of up to 10.39%.^369^ PPy has been employed as a counter electrode in DSSCs demonstrating a PCE up to 5.75%.^370^ PPy has also been utilized as a dopant material to enhance conductivity. For instance, PPy nanoparticles were recently used as a dopant for PEDOT: PSS, resulting in a high PCE of 9.48%, which was 20% higher than that of a pure PEDOT: SS-based OSC device.^371^ CP based materials are also finding applications in textile-based SCs. A recent development involved the use of PPy coated carbon fabric as a counter electrode in a textile-based DSSC, achieving a PCE of 3.86%.^372^ CP-based PPy has been employed as a catalytic layer on Ni-coated fabric to develop fabric-based DSSCs, resulting in a PCE of 3.30%.^373^ PPy-coated PANI has recently been employed as a counter electrode in a textile-based DSSC, yielding a PCE of 3.8%.^374^ These findings highlight the potential of CPs in textile-based SCs.

### Active/Absorbing Layer

**Figure 9:**
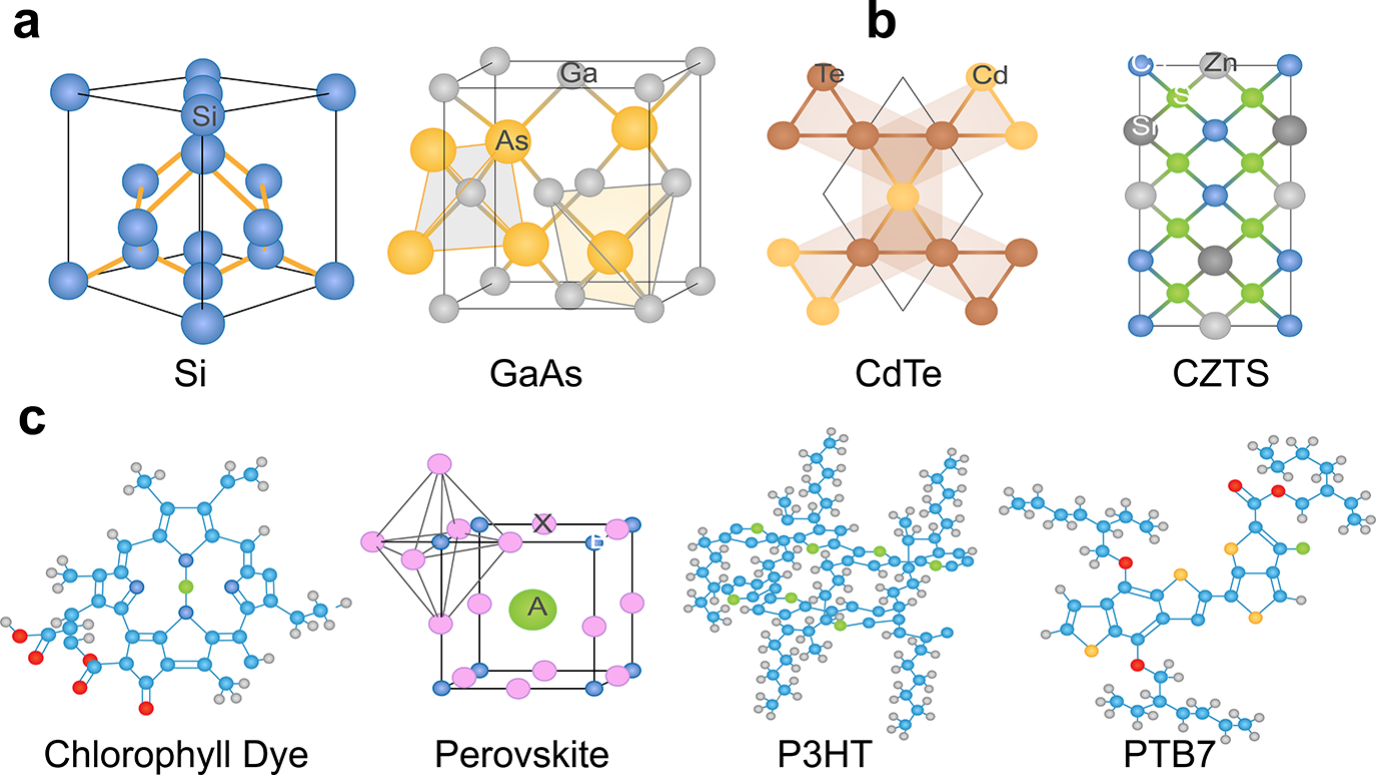
Chemical and crystal structures of commonly employed absorbing/active materials. (a) First-generation, (b) second-generation, and (c) third-generation SCs.

Active layers, also known as absorbing layers, consist of photoactive materials and play a crucial role in the photovoltaic process. This layer is where the photons from the sun energize the electrons, creating electron–hole pairs. The generated charge carriers are then separated by an electric field established in the depletion layer. After passing through a load, these charges can recombine through electrodes.^138,387^Figure 9a illustrates the general chemical structure of first-generation SC absorbing/active materials. Silicon has been a game-changer in the field of solar energy harvesting and has played a pivotal role in the development of SCs technology.^388,389^ The journey of silicon SCs has seen steady progress starting with the initial achievement of 1% PCE and currently commanding a 95% market share.^390,391^Figure 9b showcases the chemical structures of the most prevalent photoactive materials used in second-generation SCs, including cadmium telluride (CdTe), copper zinc tin sulfide (CZTS), and amorphous silicon (a-Si).^392−394^

Active materials in third-generation SCs encompass various types of materials, including perovskites (organic/inorganic), dyes, quantum dots and organic polymers, among others. Figure 9c illustrates the chemical/crystal structures of some common third-generation active materials. In OSCs, the active layers were initially formed of a single-component organic semiconductor layer, resulting in low performance due to the limited excitons dissociation and charge transfer in such materials.^395^ However, in recent decades, several electron donor materials, particularly polymers and small compounds, have been synthesized. Poly(3-hexylthiophene) P3HT is one of the oldest and most common polymer materials employed in OSCs, showing excellent photovoltaic capabilities. Its absorption spectrum (500 to 650 nm) is however still relatively narrow for achieving a high PCE. Different composites of P3HT with other materials such as ([6,6]-phenyl-C61-butyric acid methyl ester) PCBM with P3HT,^396^ etc., are being studied to broaden the absorption range. PCBM, one of the most effective fullerene derivatives, has also been studied as an electron-accepting material due to its favorable photovoltaic capabilities.^397^

### Charge Transport Layer

The charge transport layers are situated between the electrode and the active layers in thin-film SCs. These layers play a crucial role in facilitating charge carrier extraction and recombination processes, thereby enhancing the electrical performance of thin-film SCs.^398−400^ The choice of materials for the transport layer has a significant impact on the efficiency of the SC. As there are two types of charge careers, negative (also known as an electron) and positive (also known as a hole); two types of charge transport layers are primarily used: the (i) electron transport layer and the (ii) hole Transport layer.

### Electron Transport Layer

The electron transport layer (ETL) plays several crucial roles in thin-film SCs. Its main functions include collecting and transporting the photoelectron carriers, as well as functioning as a blocking layer to prevent the hole recombination at the early stage of generation and enhancing the efficiency of SCs.^401,402^ Titanium Oxide (TiO_2_) is a widely used material for the ETL due to its wide band energy range, with a conduction band maximum (CBM) of 4.4 eV and the valence band maximum (VBM) of 7.63 eV. This allows for efficient electron transfer from the perovskite layer and effective hole blocking at the interface with the active layer.^403,404^ Other commonly used ETL materials include Tin Oxide (SnO_2_), PCBM and Zinc Oxide (ZnO_2_).^405,406^ While traditional fabrication techniques for TiO_2_-based ETL require high temperatures, recent reports have demonstrated it occurring at around 100 °C.^407^ Optimizing the thickness of the ETL layer is critical to boosting SC performance.^408^ Different processes such as chemical bath coating,^409^ spin coating,^410^ spray pyrolysis, sol–gel^411,412^ and atomic layer deposition (ALD)^413^ are employed to develop ETL layers in solution process-based SCs.

**Figure 10:**
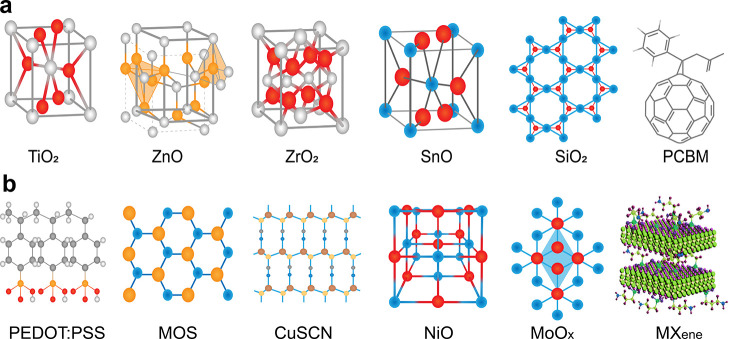
Chemical and crystal structures of commonly used (a) electron transport materials and (b) hole transport materials.

In addition to TiO_2_, other transparent metal oxides, namely zinc oxide (ZnO), indium oxide (In_2_O_3_), and tin oxide (SnO_2_), exhibit excellent optical and electrical properties, as well as significantly higher electron mobility. These alternative metal oxides have been extensively studied in the context of SCs, including PSCs, DSSCs, OSCs.^414,415^ In a recent study, a planar PSC was developed by low-temperature solution-processing, where ZnO nanoparticles were employed as an ETL achieving a PCE of 15.7%.^416^ SnO_2_ has recently been found to have considerably larger bandgap energy and substantially better electron mobility than TiO_2_.^417^ SnO_2_ has been employed as an ETL layer in PSCs and has demonstrated improved efficiency in terms of electron extraction, stability and PCE.^418^ The chemical/crystal structures of some common ETLs are illustrated in Figure 10a.

### Hole Transport Layer

Hole transport layer (HTL) materials play a crucial role in SCs, particularly in organic OSCs significantly impacting device stability and PCE.^419^ The main function of HTL is to collect and transport the generated holes toward an external electrode and facilitate their recombination after passing through load on return.^420,421^ Additionally, HTL also serves as a moisture barrier in SCs.^422^Figure 10b depicts the crystal structures of some common HTL materials. A recent study investigated the impact of the HTL layer in SCs by fabricating PSCs with and without the HTL layers. It was observed that the device without the HTL exhibited a lower generated *V*_oc_, indicating higher recombination losses at the electrode.^421^ While both organic and inorganic HTL materials are used in SCs, inorganic materials are not preferred for PSCs and OSCs due to their high-temperature requirements and costly deposition processes, despite their potential for achieving a high PCE.^423^ Materials such as CuSCNCuI, and NiOx are examples of inorganic HTLs. On the other hand, organic HTLs are gaining popularity, particularly in OSCs, due to their lower cost and ease of roll-to-roll manufacturing.^424^ Organic HTL materials commonly used in OSCs include PTTA, P3HT PEDOT: PSS, spiro-OMeTAD, among others. Recently two-dimensional materials such as MoS_2_ and, WS_2_, have also been utilized in OSCs.^420^ HTLs were implemented in PSCs in 2012, with the organic spiro-OMeTAD material being used. Since then, spiro-OMeTAD has remained a popular choice for HTLs, and its performance has been improved when combined with additives like 4-*tert*-butylpyridine (tBP).^425^ Dopant-free HTLs using alternative organic materials, such as PEDOT: PSS, are also being explored for PSCs and OSCs.^426,427^ Another widely used organic material is poly[bis(4-phenyl)(2,4,6-trimethylphenyl)amine] (PTTA) as a hole transport materials (HTM) in PSCs. Like spiro-OMeTAD, PTTA has stability issues and requires specific additives for optimal performance. Recently, mesoporous PSCs with a defect-engineered thin perovskite layer were fabricated using lithium bis(trifluoromethanesulfonyl)imide (tBp-LiTFSI) doped poly(triarylamine) (PTAA) as the HTMs, achieving a PCE of 22.1% for a 1 cm^2^ SC.^428^ However, one major drawback of organic HTLs is their stability. In addition to organic materials, there are also inorganic HTL materials available. Examples of inorganic HTMs include Cu_2_O, CuO, CuI, CuSCN, NiOx, and MoS_2_. Other materials like CuS, CuCrO_2_, MoOx, and WOx have also been investigated for HTL applications.^429^ According to recently reported articles, inorganic HTMs exhibit higher hole mobility, excellent stability, and lower cost compared to organic HTLs^420,430−433^ These advantages have led to an increased research interest in inorganic HTMs.

### Other Materials for Miscellaneous Purposes in SCs

Other materials, such as transition metal dichalcogenides (TMDs), black phosphorus (BP), phosphorene, hexagonal boron nitride (h-BN), and MXene, have been employed in various functions within SCs due to their functionalization and bandgap re-engineering capabilities.^50^ These materials have been utilized as absorber layers, charge transport layers, blocking layers, surface passivators, heterojunction components, catalysts, and as electrodes. Among these materials MXene have been extensively employed for a wide range of applications, including energy harvesting, energy storage, photonics, advanced sensors, and healthcare devices; a general crystal structure is shown in Figure 10b.^434−437^ Because of its promising electrical conductivity, transparency, flexible work function, and robust mechanical characteristics, MXene has gained popularity in the field of SCs since it was reported in 2018.^438^ MXenes exhibit semiconductor-like characteristics with a direct bandgap at the monolayer level, making them suitable to be used as an active material in flexible SCs.^439,440^ Numerous investigations have examined the adaptability of MXene in SC technology, wherein it functions as a transparent electrode, a counter electrode, as an ETL and HTL.^441−443^ These diverse uses highlight how these materials may be used in a variety of SC technologies for a range of applications.

## Fabrication Techniques for Textile-Based SCs

SCs are composed of multiple layers, requiring several distinct manufacturing steps. It is important to note that the assembly of textile-based SCs differs from that of rigid surface SCs, even though the deposition or fabrication process for different layers, such as coating and printing processes, are the same. This section mainly focuses on solution processes and techniques employed in thin-film SC technologies.

### Materials Preparation and Fabrication

One of the most critical aspects of fabricating any electronic device is the preparation of materials suitable for the fabrication process. However, certain materials, such as active/absorbing materials, ETL and HTL, are not always single substances but rather combinations of two or more materials that need to be synthesized into a composite solution. Each material requires distinct chemical and physical procedures for preparation, such as high temperature or an oxygen-free atmosphere, among others.^444−447^ Furthermore, material preparation in accordance with the manufacturing procedure is an important factor to consider. Because of the varied viscosity and surface tension requirements, the procedure for producing screen printable ink differs from that of spray coating. To achieve maximum performance and compatibility with the selected process, each manufacturing method demands certain changes in material preparation. After preparing and obtaining the materials, the next step involves the fabrication and deposition of those materials. The next section will discuss the various fabrication processes, along with their benefits and drawbacks, in terms of textile-based SCs.

### Spinning

**Figure 11:**
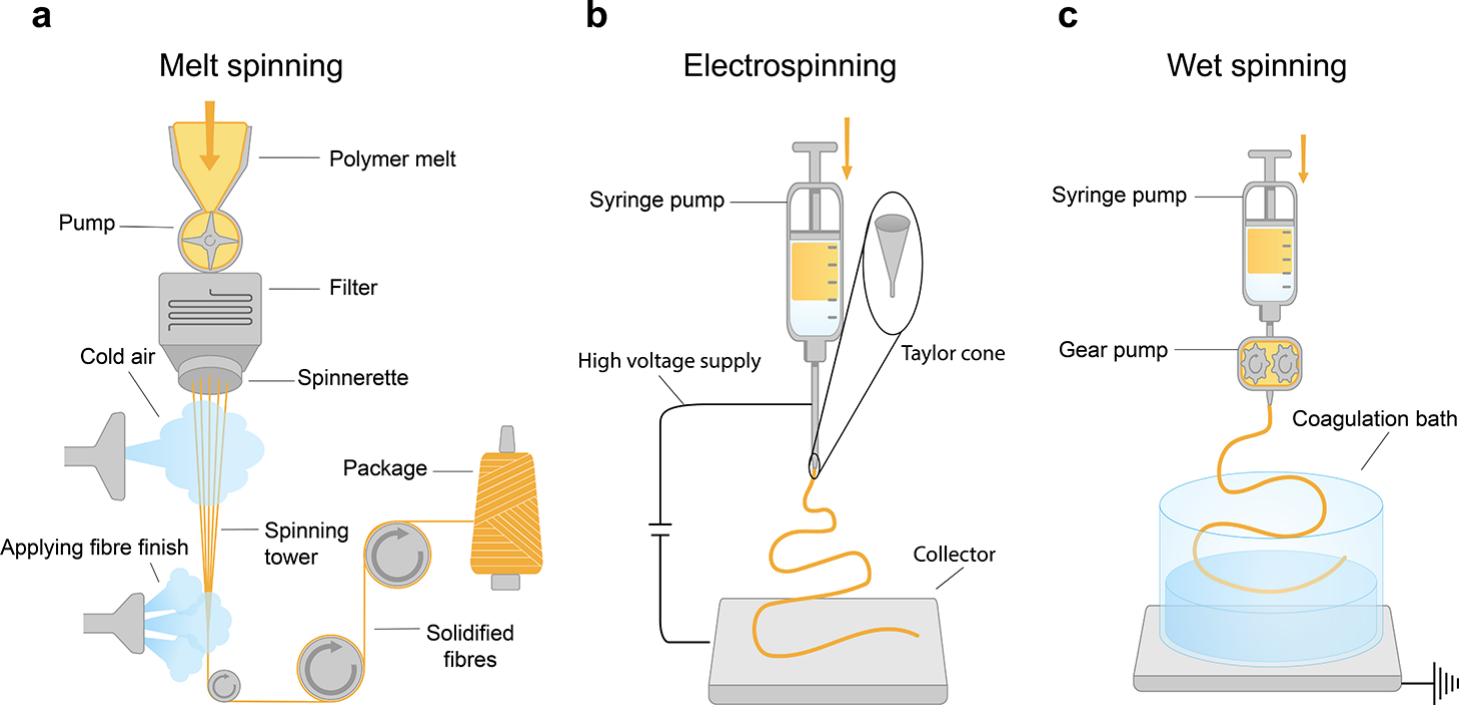
Spinning techniques for photovoltaic textile fabrication: (a) melt spinning, (b) electrospinning, and (c) wet spinning.

Conductivity is a fundamental characteristic that must be achieved in the development of e-textiles. There are several known methods used to achieve electrical conductivity or other functionalities on a textile substrate. Spinning is one useful process that is gaining popularity because of its ability to maintain the original form and breathability of the final fabric. In the spinning process, liquid polymeric filaments are extruded and continuously drawn while being solidified to create a continuous synthetic fiber.^448^ There are three main categories of spinning techniques: wet spinning, melt spinning, and dry spinning,^449^ as shown in Figure 11.

Figure 11a illustrates the melt spinning process, where polymeric pellets or microbeads are introduced into a chamber and melted. The liquid is then filtered and pumped through a nozzle to cool and solidify it. Fibers are formed from a melted and cooled polymer. If conductive fibers are desired, materials such as graphene and others are added to the polymeric materials during the processing.^450,451^ Melt spinning is limited to polymers that are thermally stable well above their melting points, and the hardening of fiber occurs during the drawing process as they are cooled below their glass transition temperature. Electrospinning and wet spinning are common solvent-based spinning processes, as shown in Figure 11b and c, respectively. In these processes, the fiber is extruded into a nonsolvent medium. The solvent-based methods are preferred to produce conductive wires due to their low processing temperature. The wet spinning method is effective for producing fibers with various cross-sectional diameters.^452−454^

### Coating

**Figure 12:**
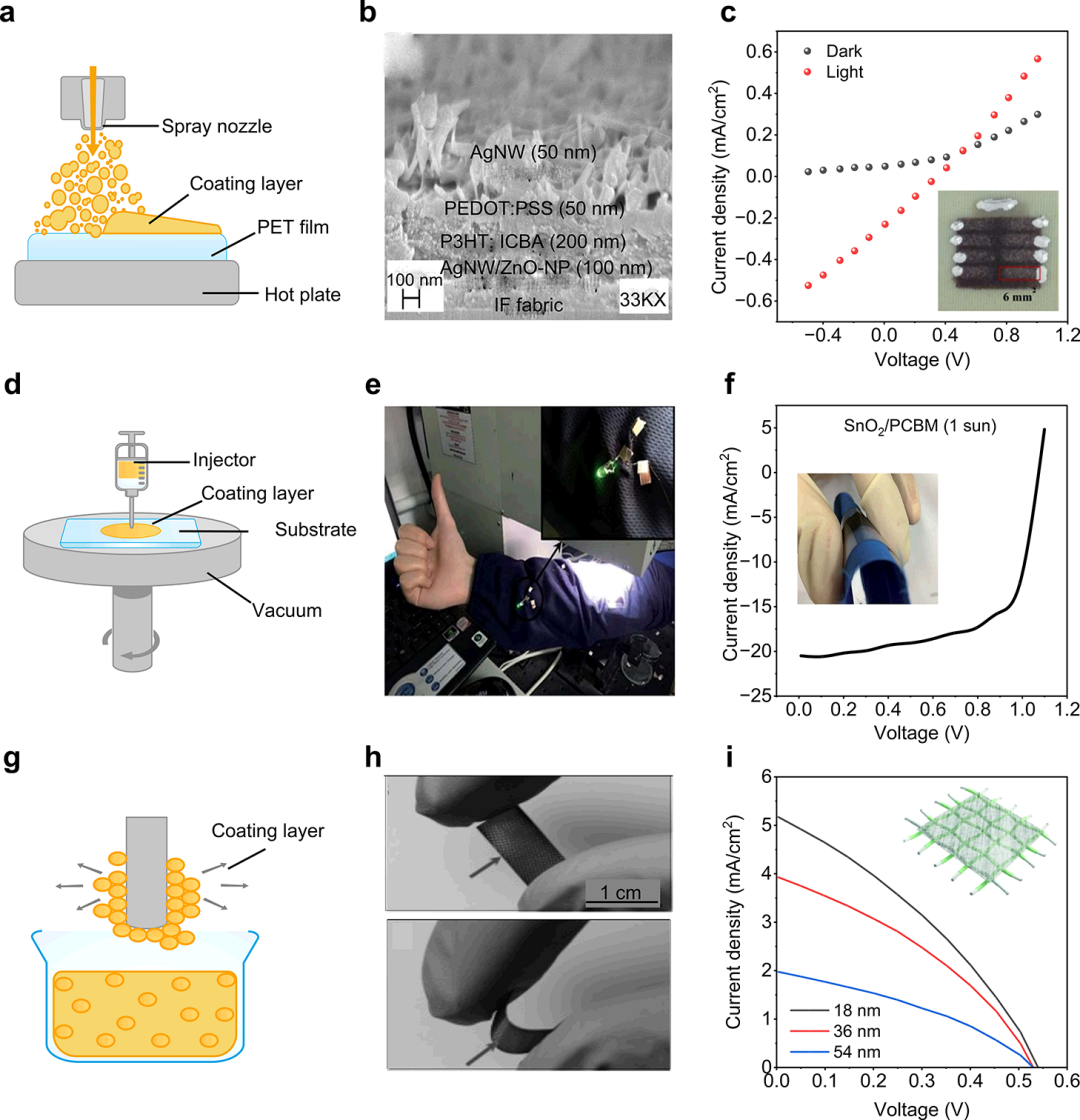
Coating techniques for photovoltaic textile fabrication. (a) Schematic of the spray coating system. Fully spray coated OSC device (b) SEM image and (c) the performance curve in both light and dark mode with an original photograph (inset) of the fabricated device. Reprinted with permission from ref (457). Copyright 2016 The Royal Society of Chemistry. (d) Schematic of a spin coating system. (e) A spin-coated wearable PSC device under sun simulator, while glowing an LED upon exposing to light and (f) the photograph of the spin-coated PSC device (inset) along with the corresponding IV curve under 1 sun condition. Reprinted with permission from ref (294). Copyright 2017 The Royal Society of Chemistry. (g) Schematic of dip coating process, (h) the photographs of the PSC device where the active layer was dip-coated and (i) the performance curves of the fabricated PSC with respect to different thicknesses of the active layer. Reproduced with permission from ref (462). Copyright 2014 Wiley-VCH Verlag GmbH and Co. KGaA, Weinheim.

The coating process is one of the common and straightforward techniques used for layer deposition in nanotechnology applications.^6^ Techniques such as spray coating, dip coating and spin coating are widely employed in the field of textile-based and flexible devices. Spray coating involves propelling the ink of the desired material through a nozzle and spraying it onto a surface or substrate using an air pressure pump, as depicted in Figure 12a.^455,456^ Recently, spray coating has been employed to directly fabricate OSCs onto textile substrates. The process involves initially applying an interface material to smoothen the textile surface, followed by the sequential deposition of different layers using spray coating. Figure 12b depicts corresponding cross-sectional SEM images of the fabricated OSC, while Figure 12c presents a photograph of the fabricated OSC device accompanied by its performance curve.^457^ Spray coating is considered a repeatable and efficient method for applying thin functional layers onto fabrics. Recently a textile-based PSC was fabricated using the coating technique for the PU interface layer. Planar textile-based- PSCs have achieved a PCE of 5.72% and the devices demonstrated outstanding flexibility under ambient environments.^458^

Another highly applicable coating technique is spin coating. Spin coating is a simple and cost-effective method for depositing thin and homogeneous film layers on flat surfaces. In this process, a micropipette or a syringe is used to drop-cast a predetermined volume of dispersion into the spin coater to spin the substrate at high speed (up to 10,000 rpm), which allows the fluid to be distributed throughout the substrate using a centrifugal force, as shown in Figure 12d.^459^ Recently, except for the ETL layer, an entire textile-based PSC device has been fabricated using a spin coating technique. Figure 12e shows the photograph of the device being worn on a hand while lighting an LED upon exposure to a sun simulator. Figure 12f depicts a characteristic IV curve under a 1 sun condition, along with a photograph of the fabricated device.^294^ In another study, the spin coating was utilized for the entire device fabrication of an OSC device in a 9 cm × 9 cm array on a glass substrate, achieving a PCE of ∼14%.^460^ These findings highlight the flexibility of spin coating in nanofabrication and nanotechnology. Although the spin coating technique is not directly suitable for textiles due to their rough surfaces and high absorbency, the surface of textiles can be modified using interface materials to make them smoother and more uniform before coating.^461^

Another common method, “Dip coating,″ involves immersing the substrate/textile components into the coating dispersion, as shown in Figure 12g. In a recent fabrication of a PSC device, the active layer was coated using a dip coating process. A photograph of the device in a straight and bent shape is shown in Figure 12h. The performance curve of the fabricated PSC device with different thicknesses of active materials is shown in Figure 12i, where the highest performance was achieved with an 18 nm thick layer.^462^ In another PSC fabrication, the ETL layers (SnO_2_) were coated using four cycles of dip coating and they obtained a PCE of 3.2%.^463^ These results demonstrate the advantages of employing dip coating, as it is an easy and cost-efficient technique.

### Printing

Printing, in its most basic sense, refers to the act of transferring a pattern, such as an image, text, or any other kind of pattern, from one surface (like a page or piece of cloth) to another. In the field of nanotechnology, printing is employed to deposit layers or patterns onto a substrate for the manufacturing of printable devices.^464^ When it comes to fabricating wearable devices, screen printing and inkjet printing are the two techniques that are most commonly utilized.^7,465,466^

### Screen Printing

**Figure 13:**
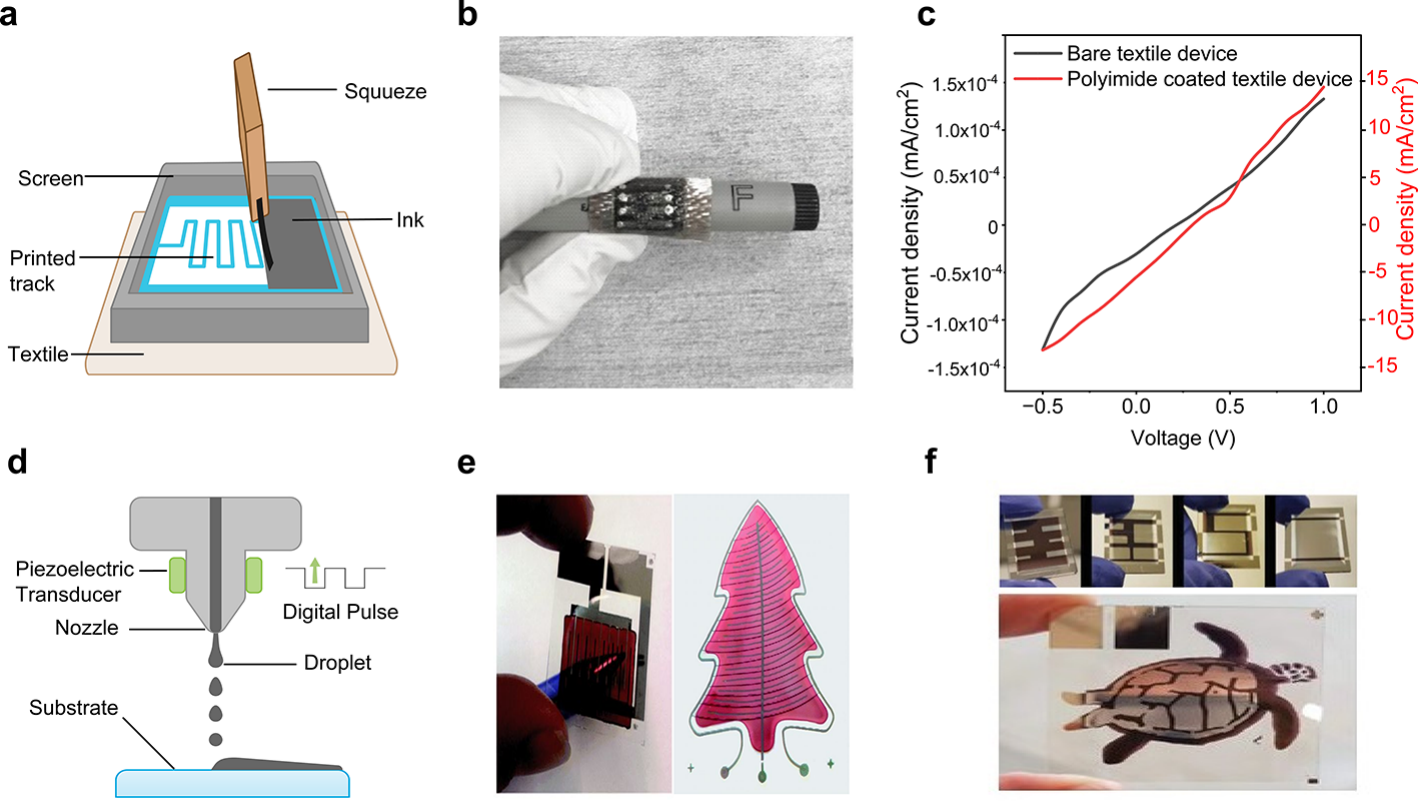
Printing techniques for photovoltaic textiles fabrication. (a) Schematic of a screen printer and its different parts, (b) a photograph of screen printed DSSC-based on glass fabric and (c) the corresponding IV curve for two different devices with and without an interface layer. Reprinted in part with permission under a Creative Commons CC-BY License from ref (469). Copyright 2019 Springer Nature. (d) Schematic and different parts of inkjet printer. (e) Photographs of the inkjet-printed OSCs, where the shape OSC is fabricated in the shape of a Christmas tree. Reprinted in part with permission under a Creative Commons Attribution 3.0 Unported License from ref (473). Copyright 2015 The Royal Society of Chemistry. (f) The shape of an inkjet-printed sea turtle on an ITO Glass substrate. Reused with permission from ref (474). Copyright 2019 WILEY-VCH Verlag GmbH and Co. KGaA, Weinheim. These works demonstrate the capabilities of inkjet printing to be employed for a variety of fine patterns.

The method of screen printing involves a stencil process that transfers ink onto a surface or substrate. Figure 13a illustrates the setup for screen printing, highlighting the different parts involved. When it comes to textile-based SCs, screen printing has been employed either fully or partially by various researchers.^467,468^ For example, a solid-state DSSC on a textile fabric was recently developed using both screen printing and spray coating techniques. A photograph of the fabricated device is shown in Figure 13b and c showing the performance curves of two devices with and without the interface materials.^469^ Similarly another textile-based DSSC was fabricated employing screen printing for printing the polyurethane (PU) materials as a interface layer to smoothen the surface. Additionally, screen printing was also employed for the electrode (silver bottom electrode) and TiO_2_ as the ETL layer.^470^ Screen printing is a simple and cost-effective technique in terms of technology and can be used on a wide range of materials. However, the process may be time-consuming due to the need to prepare a pattern and for careful cleaning of the screen after printing.

### Inkjet Printing

Inkjet printing is one of the emerging technologies utilized especially for fabricating wearable devices.^471^ The print head in an inkjet printing method consists of multiple minute nozzles, also known as jets. When the substrate passes in front of the print head, the nozzles digitally designed patterns or images onto the substrate (see, Figure 13d). Inkjet printing has been utilized in a variety of applications such as sensors, supercapacitors, and SCs.^7,465^ Inkjet printing has been utilized for the full or partial fabrication of SC devices.^472^ Recently, fully inkjet-printed OSCs were successfully fabricated using an industrial-scale inkjet printer equipped with 512 nozzles printheads. Figure 13e presents a photograph of two fabricated OSC devices, one of which is in the shape of a Christmas tree.^473^Figure 13f shows photographs of the OSC devices, where one of them is fabricated in the shape of a turtle employing inkjet printing techniques.^474^ These demonstrations highlight the versatility of inkjet printing, as it enables the fabrication of various intricate structures without the need for masks.

Despite inkjet printing being a popular and material-effective approach for device and solar fabrication,^475,476^ there are some limitations to consider. Developing fine layers with an inkjet printer requires smooth surfaces like a sheet of paper. A cartridge also needs changing every time for printing different materials, increasing the cost compared to other printing methods. Finally, textiles do not have a smooth surface, and they tend to absorb ink more, making direct inkjet printing on textile substrates very challenging. To address this, the surface of textiles can be modified to have a smoother appearance by applying an interface material. However, this modification may negatively impact the light absorption and alter the original fabric appearance.

## Assembly of Textile-Based SCs

In this section, we will provide a detailed overview of the assembly of textile-based SCs. There are three main methods utilized for the fabrication of these SCs: stacking of prefabricated SCs, the direct layer-by-layer fabrication method, and yarn intersections. Each of these will be discussed in detail.

### Prefabricated Solar Cell Stacking

**Figure 14:**
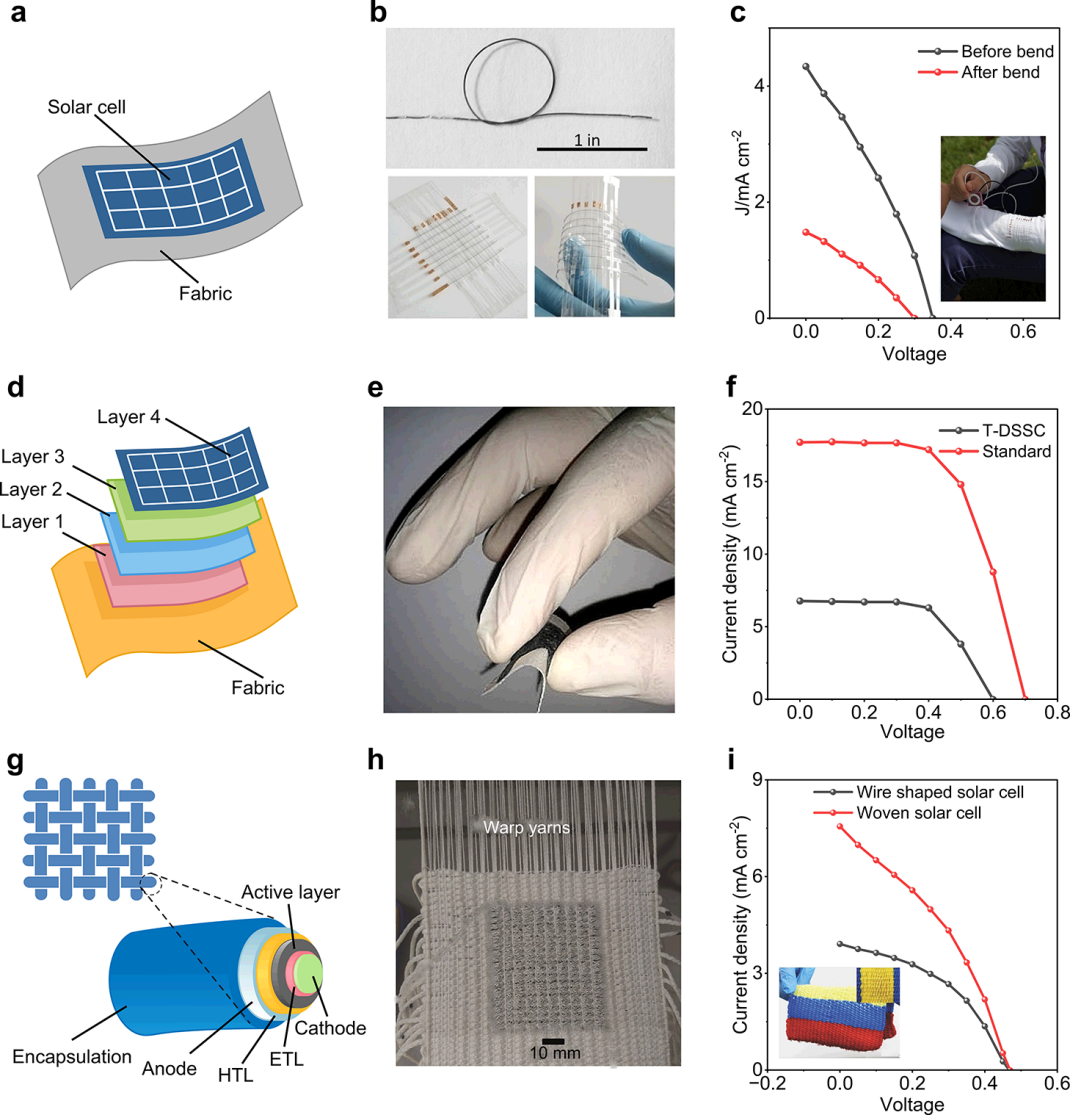
Assembly methods for photovoltaic textiles. (a) Schematic of a preprepared SCs stacking on a fabric, (b) flexible SCs wire interwoven, and (c) the IV curves before and after bending along with the photograph, powering an MP3 device under sunlight. Reproduced with permission from ref (478). Copyright 2014 WILEY-VCH Verlag GmbH and Co. KGaA, Weinheim. (d) Schematic of layer-by-layer process of a textile-based SC. (e) A photo of the layer-by-layer fabricated textile DSSC and (f) their IV curves in comparison to the standard device, respectively. Reproduced with permission from ref (484). Copyright 2017 Elsevier. (g) Schematic of SC’s yarns, interwoven with zoomed cross section and (h) A photograph of a SC yarn-based fabric. Reproduced with permission under a Creative Commons CC-BY License from ref (130). Copyright 2019 John Wiley and Sons. (i) The IV curves of a yarn-based SC in both yarn and textile shape, along with a photograph of the device (inset). Reproduced with permission from ref (485). Copyright 2015 WILEY-VCH Verlag GmbH and Co. KGaA, Weinheim.

The practice of fixing one layer or device over another is known as layer stacking (see Figure 14a). The direct attachment or integration of SCs to textile substrates is an old and well-established technique. This is a simple and efficient way to build Textile-based SCs and offers several advantages. For example, it saves the textile from high-temperature processing, which can potentially burn or damage the materials. Numerous researchers have described the incorporation of prefabricated SCs into fabrics. Common methods employed for stacking include stitching, hot-melting and wet- transferring etc.^217,477^ For example a SCs integrated winter jacket produced by Maier Sports in collaboration with the Institute for Physical Electronics at the University of Stuttgart and delivered up to 2.5W of power.^217^ Another recent development includes a PSC device in the shape of wires, which were weaved into flexible clothing to provide a lightweight and flexible power source. Figure 14b shows original photos of SCs in flat and bent forms, while Figure 14c shows the IV curves before and after bending along with a photo while powering an MP3 device under sunlight.^478^ Polymer SCs on woven textiles were also developed using a free-standing wet transfer method, achieving a PCE of 2.9%.^479^ In a study, the hot-melt process was utilized to integrate an ultraflexible and thermally stable OSC into textiles, demonstrating a PCE of 10% without degrading the performance.^480^ Using the stitching mechanism, an OSC device was vertically transferred onto a textile, resulting in an enhanced PCE of approximately 1.8%.^266^ Although the direct transfer is simple and effective in maintaining the functionality of SCs, it hampers the appearance of the fabrics and makes them less comfortable to wear. It also affects the washability of the textiles. Therefore, researchers are favoring alternative methods such as the layer-by-layer approach for preparing textile-based SCs.

### Layer-by-Layer Fabrication

The “Layer-by-layer” method is considered a promising approach to preserve the integrity and original state of the textiles in terms of their shape and comfort level (see Figure 14d). By developing a structure in layers, strong contact is established, resulting in excellent stability and mechanical durability against disturbances.^481^ The layer-by-layer approach is considered a prominent approach for scalable textile-based SCs production.^482^ Coating and printing are the primary manufacturing processes used in the fabrication of textile-based SCs, as discussed in previous sections.^24^ A research team used combined printing and coating techniques to develop an entirely textile-based OSC, although its PCE was significantly lower than that of other substrates.^483^ Additionally, a DSSC device fabricated on a polyester fabric substrate using the layer-by-layer approach is presented, maintaining flexibility and achieving a PCE of 2.5% (Figure 14e,f).^484^

### Yarns Intersection

Although stacking and direct layer-by-layer approaches are straightforward and effective for building textile-based SCs, both mechanisms can significantly compromise the breathability of the fabric, leading to wearer discomfort. Additionally, the presence of top electrodes, adhesive layers, and stitching can potentially impede the absorption of light energy, resulting in a decrease in the SC performance.^353^ These limitations have prompted researchers to explore alternative approaches. Weaving and knitting using active yarns, such as photoactive fibers or electrode fibers, have emerged as promising methods to overcome the drawbacks of stacking. The surface topology and bare regions of photoactive fibers allow these SCs to maintain breathability and effectively absorb solar energy from diverse light incidence angles. Figure 14g presents a schematic of a SC yarn. Figure 14h showcases a picture of a fabricated yarn-based SC textile, which was developed using silicon SC-embedded yarns connected by fine coper wires.^130^ Recently, wire-based DSSCs were developed using polybutylene terephthalate (PBT) wires as a substrate, with each wire achieving a PCE of 1.3%. Figure 14i shows a photograph and IV curves of both single fibers and yarns in a textile form of the SCs.^485^ The yarn-based approaches demonstrate the potential for integrating SC functionality into textiles while preserving breathability and optimizing light absorption due to shadows between interwoven yarns.

## Performance of Textile-Based SCs

Researchers in the field of renewable energy have recognized the potential of integrating SCs into textiles due to their exposure to sunlight and their prevalence in everyday human life. While fabricating silicon-based SCs directly on textile substrates is challenging, various techniques have been developed to embed SCs into textiles. One recent study, for instance, integrated silicon SCs into textiles using two approaches: a tessellation design for stiff folding and textile-based deformable metallic connections. The performance of the foldable tessellated textile embedded with silicon SCs was superior to that of a traditional module.^486^ Recently, eight monocrystalline SCs were encapsulated with functional synthesized fabric materials using an industrial textile lamination technique. These SCs demonstrated reliable laundering durability, meeting the ISO 6330:2012 standards. After 50 laundering cycles, five of the eight devices exhibited no change in PCE, while three devices experienced a decrease in PCE performance ranging from 20–27%.^487^ Besides, the first-generation SCs and second-generation thin-film SCs have also been explored for textile applications. Thin-film SCs based on materials such as CIGS (copper indium gallium selenide) and amorphous silicon have shown promise in integrating with textiles.^130,488,489^ The potential applications of SCs in wearable and functional fabrics are fascinating. While the efficiency of first- and second-generation SCs for powering wearable devices is noteworthy, their large and rigid structure affects the fabrics’ originality and breathability.

With the emergence of the latest SC technologies, particularly third-generation SCs that offer enhanced flexibility and solution processability, there has been a renewed interest in textile-based solar energy harvesting. These advancements, as highlighted in various studies,^458,485,490^ have positioned third-generation SCs as sustainable options for researchers to explore in the development of textile-based SCs. Consequently, this Review further categorizes the performance of textile-based SCs based on the specific type of SCs, focusing on the potential of third-generation SC technology, such as DSSCs, PSCs, OSCs, and QDSCs.

### Textile-Based DSSC Performance

**Figure 15:**
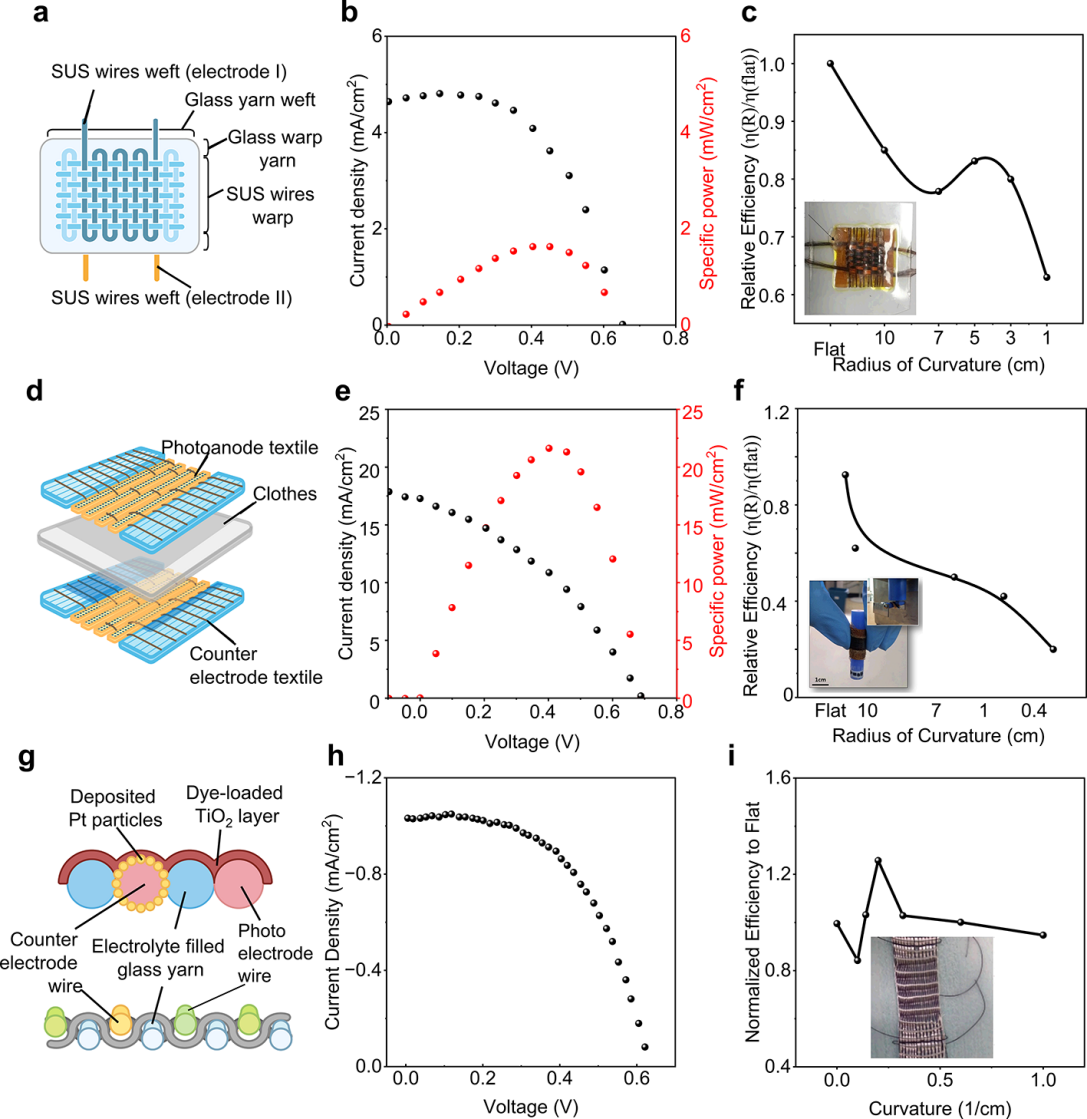
Performance evaluation for DSSC-based photovoltaic textiles. (a) Schematic of a proposed 3D textile-based DSSC. (b) the electrical performance of the device: current density and specific power. (c) the relative efficiency with respect to the radius of curvature in centimeters along with a photograph of the fabricated textile-based DSSC. Reprinted in part with permission under a Creative Commons CC-BY License from ref (492). Copyright 2019 Springer Nature. (d) A schematic of a recently developed textile-based DSSC, (e) the current density and specific power of the fabricated device and (f) a photograph of the device in a bent shape along with the relative PCE with respect to the radius of curvature. Reprinted with permission from ref (493). Copyright 2014 Springer Nature. (g) A schematic of another recently investigated textile-based DSSC, (h) the current density curve of the device, and (i) a photograph of the fabricated device along with the normalized efficiency as per radius of curvature. Reprinted in part with permission under a Creative Commons CC-BY License from ref (218). Copyright 2016 Springer Nature.

Textile-based DSSCs have been the topic of extensive studies over the past decade because they are lightweight, flexible, sustainable, possess low-cost efficiency, are easy to process, and the potential for industrial manufacturing techniques such as coating, printing, and other similar techniques.^165^ A flexible DSSC based on a solid polymer electrolyte was developed using a platinum-coated stainless-steel wire as the counter electrode. ZnO-coated photoelectrode (PE) and the counter electrode (CE) were woven together to produce a satin weave structure (10 × 10 wires). Under typical lighting conditions (AM 1.5 G), the developed flexible DSSC achieved a PCE of 2.57%, with *V*_oc_ of 0.45 V.^491^ A textile DSSC device was fabricated using poly(butylene terephthalate) (PBT) polymers, interwoven in two distinct patterns and filled with a solid dye. The developed textile DSSC attained a PCE of 1.3% under a standard illuminance (AM 1.5 G) for a single cell unit and *V*_oc_ of 4.6 V.^213^ A three-layered textile DSSC was woven concurrently on a Jacquard loom, utilizing stainless steel wires as electrode bases and spacers to create a basket-like structure. Figure 15a provides a schematic of the proposed textile DSSC, where the PE was developed using TiO_2_, which was applied to the stainless-steel woven electrode using float printing and sintering techniques. For the CE, platinum was coated with an activated carbon paste. Figure 15b displays the IV performance curve and the power density curve. Despite an initial PCE of 1.7% and *V*_oc_ of 0.64 V, the fabricated device experienced a 20% decrease in PCE within 1 day and a 50% decrease within 7 days. Additionally, the flexibility of the fabricated textile DSSC was analyzed (Figure 15c) by studying its performance curve at different radii of curvature. The photograph (inset) of the device is also shown in Figure 15c. A significant decline in PCE was observed as the radius of curvature decreased from flat to 1 cm.^492^

The wire interlacing-based textile DSSCs show good electrical performance results; however, the mechanical stability and output consistency have not been prominent to date. Researchers have recently determined the fabrication of direct layer-by-layer textile DSSCs that offer enhanced flexibility. In a recent investigation, a flexible sandwiched-type DSSC textile (as shown in Figure 15d) was investigated. A polyamide interface layer was coated on a glass fiber surface to smooth the roughness, followed by the printing of an Ag bottom electrode layer. Spray pyrolysis was employed as the method to apply a TiO_2_ compact layer (CL) onto the fabric, preventing the occurrence of a short circuit between the counter electrode and the solid-state electrolyte. The PE was formed by annealing the TiO_2_ layer after it had been placed on top of the TiO_2_ CL, and the solid-state electrolyte solution was added to dye-sensitize the PE. A PEDOT: PSS layer was then spray-coated to serve as the hole transport layer (HTL). Though the SC achieved a PCE of only 0.4%, the research work introduced the development of textile DSSCs through a direct layer-by-layer process. The electrical performance curves of the fabricated textile DSSC is shown in Figure 15e. A photo of a fabricated device, along with the impact on PCE with respect to the radius of curvature is shown in Figure 15f.^493^ In other research, a PCE of 7.13% was reported, where aligned MWCNT sheets were twisted onto rubber fibers using CVD to construct the counter electrode (CE) for a textile DSSC. A functional electrode in the shape of a spring was made using titanium wire with TiO_2_ nanotubes grown in a perpendicular direction. A yarn-type DSSC was developed by winding it around a flexible MWCNT fiber.^493^ Yarn-based textile DSSCs have garnered significant attention in various research endeavors. For instance, Figure 15g presents a schematic of a recently developed DSSC yarn. The fabricated yarn-based textile DSSC demonstrated a *V*_oc_ of 0.62 V and a *J*_sc_ of 1.04 mA/cm^2^, as depicted in Figure 15h. The flexibility of the device was also examined, and the results depicted in Figure 15i indicate minimal changes in PCE as the radius value decreases, thereby highlighting the high flexibility of the developed textile DSSC.^218^ The outcomes of some recent research work on textile and fiber-based DSSCs are summarized in Table 2.

**Table 2:** Performance of Some Recent Textile-Based SCs

Type	Substrate	*J*_sc_ (mA/cm^2^)	*V*_oc_ (V)	FF (%)	PCE (%)	Stability/Durability	Wearability	Ref
DSSCs	Poly/cotton textile	36.56	0.3	25	2.78	_	The device comprises both flexible fabric and rigid glass parts	(510)
	Polyester fabric	12.4	0.7	72	6.26	Decline in PCE over time, indicating an 18% reduction after 4 weeks.	Bending the CE at various angles showed no significant changes in resistance. But overall the device consists of a FTO coated glass as well	(511)
	Polyester fabric	11.92	0.69	69	5.69	CE exhibited stability, showing no notable resistance changes across varying bending angles and cycles.	Devices consist of both flexible polyester fabric and rigid glass	(512)
	Cotton fabric	14.75	0.66	71	6.93	The stability of the graphene-coated fabric remained consistent across various bending angles, a resistance change of less than 1.5%.	Consists of both fabric and rigid glass	(339)
	Cotton/Silk	15.59	0.67	47	5	Capable of enduring thousands of deformation cycles.	Durable, highly flexible, and stretchable, with a bending curvature radius of 4 mm	(493)
	Cotton fabric	9.6	0.65	52	3.3	_	Consists of flexible cotton fabric and rigid glass	(373)
	Glass fiber textile	10.24	0.73	54	4.04	The device maintained a stable PCE up to 8 weeks.	Remains functional even when bent around a 5 mm radius rod	(470)
		5.1	0.79	27.4	1.1	Remains stable for over 7 weeks.	Flexible substrates such as glass fiber and PEN-ITO was utilized	(170)
		5.2	0.31	25	0.4	Stable for 2 weeks with minimal PCE reduction in an open-air environment without encapsulation	Highly flexible and bendable	(469)
	Flexible Plastic	12.03	0.76	79	7.29	_	_	(513)
	CNTs Yarn	10.06	0.64	45	2.94	Found to be stable without any packaging for several hours	Can be woven as a fabric to make textile	(354)
		8.32	0.67	71.79	4	Found to be stable for more than 35 days.	Wearable, maintained 90% PCE for 600 bending cycles	(514)
		19.43	0.73	71	10	PCE reduced by 18% after 2000 cycles at 90-degree bending.	Maintained 86% under bending from 0 to 180 deg	(515)
	Carbon fiber	15.3	0.68	68	7.03	_	_	(516)
	Rubber yarn	16	0.71	61	7.13	Maintained 90% output after stretching 30% 50 times.	_	(517)
	Pt wire	12.34	0.69	74	6.29	_	Bent 1 cm, resulting in a PCE change from 1.0 to 0.85	(518)
	Ti wire textile	7.89	0.53	64	2.7	_	_	(519)
	Stainless steel wire	20.02	0.45	28	2.57	_	Woven structure and bendable	(491)
		4.63	0.64	56	1.7	Specific power decreased 50% in 7 days	Wearable device made with Jacquard weaving machine	(492)
PSC	Polyester Textile	12.91	0.89	51	5.72	Device retains 83% of initial PCE after 300 h.	Wearable, as fabricated on a textile substrate	(458)
	polyethylene naphthalate Textile	20.53	1.06	66	14.3	Maintained 70% of initial PCE in ambient environment for 425 h.	Flexible and wearable	(294)
	CNT	8.75	0.615	56.4	3.03	Stable for more than 96 h in ambient conditions.	Excellent flexibility, enduring over 1000 bending cycles without degradation	(520)
		2.165	0.82	35.3	0.631	_	_	(498)
	Stainless Steel wire	10.2	0.664	48.7	3.3	Maintained stable PCE (95%) upon 50 bending cycles.	Bendable in various shapes with negligible reduction in output	(200)
	Ti wire	11.23	0.67	58	5.3	_	_	(496)
		_	0.85	_	7.1	Maintained 90% of the initial results upon 400 twisting cycles.	Can be woven into a textile	(521)
		11.97	0.731	44	3.85	Over 93% PCE retained after 50 bending cycles.	Bendable	(497)
OSC	Poly/cotton Fabric	6.05	0.54	37	1.23	Lost photonic functionalities after ten cycles at 2.5 cm radius.	Bendable	(483)
	Integrated to Textile	2.15	0.769	70.8	8.7	Found to be stable after bending 100 cycles of radius 2.5 mm.	Flexible and wearable	(131)
	Gold based textile	13.11	0.57	24	1.8	To some extent found to be durable and reliable under mechanical stress.	Wearable and integrated with clothing	(266)
	Graphene sheet	8.14	0.57	54.5	2.53	Found to be stable with 5% degradation in PCE after 8 days.	_	(522)
	CNT yarn	8.1	0.55	50	2.30	PEC dropped from 2.26% to 1.77% within the first 5 days.	Found stable upon bending to 45 and 90 degrees	(523)
	Stainless steel wire	16.6	0.55	50	_		Flexible and wearable	(508)
	Ti Mesh	5.2	_		1.08	More than 80% PCE retained after 10 days.	Flexible and wearable	(462)
	Ti and TiO_2_ wire	0.98	0.42	36	0.15	Found to be stable with no change in output in 5 days.	Showed high flexibility and ease of weaving	(524)
	TiO_2_/Ti wire	9.06	0.52	38	1.78	Maintained over 70% PCE in air after 16 days.	Flexible and wearable	(478)

### Textile-Based PSC Performance

**Figure 16:**
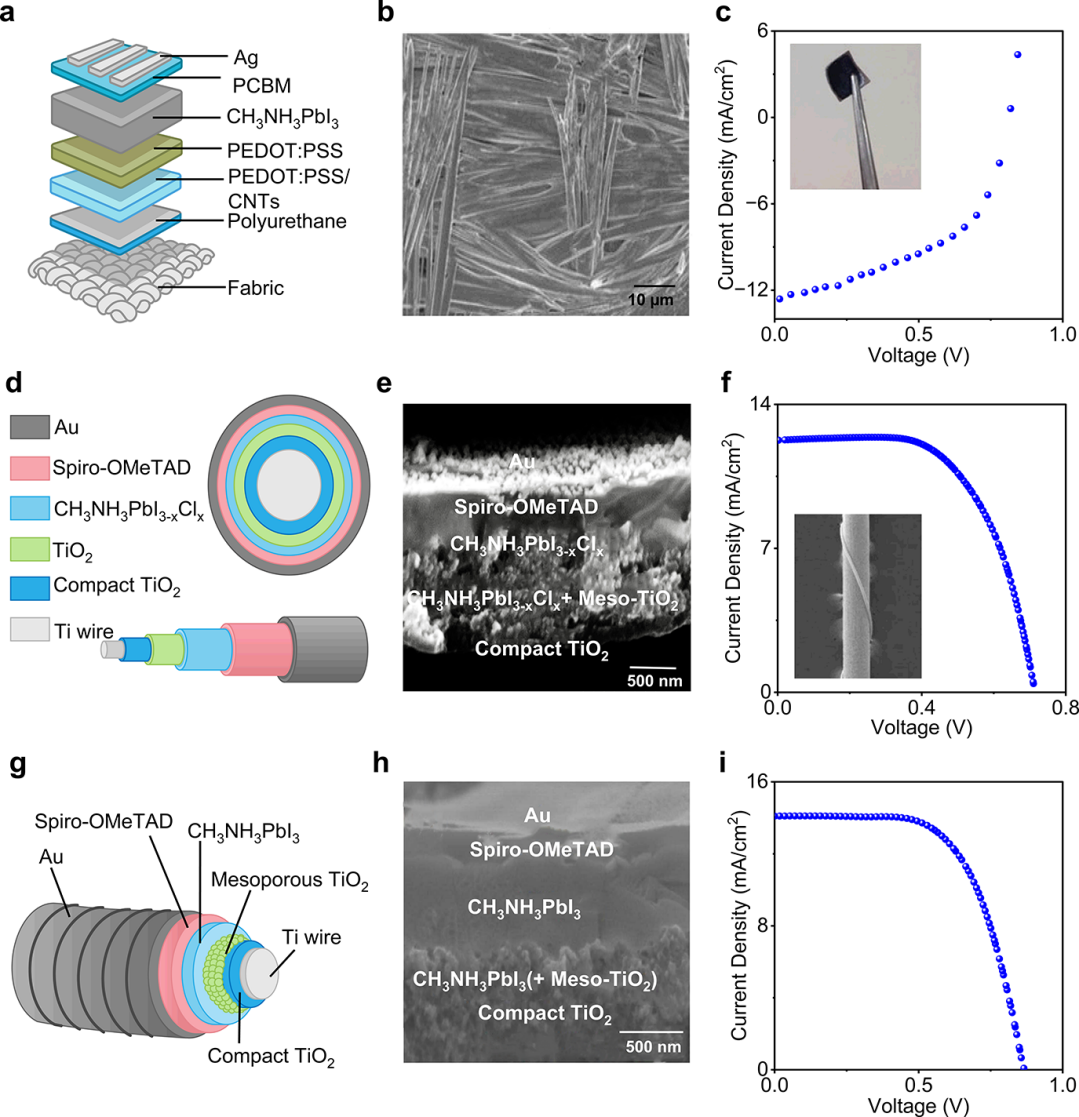
Performance evaluation for PSC photovoltaic textiles. (a) Schematic of recently developed textile-based PSC along with (b) the SEM image of perovskite layer and (c) the current density of the fabricated textile PSC as well as a photograph of the fabricated device. Reproduced with permission from ref (458). Copyright 2018 Elsevier. (d) Schematic of a fiber-shaped PSC along with (e) the corresponding cross sectional SEM image of the device and (f) the electrical performance of the corresponding fiber shaped PSC and optical image (inset) of the device. Reproduced with permission from ref (496). Copyright 2016 The Royal Society of Chemistry. (g) Schematic of a fiber shape textile PSC, (h) corresponding SEM image with indication of different layers, and (i) the electrical performance curves. Reproduced with permission from ref (205). Copyright 2018 The Royal Society of Chemistry.

The discovery of organic–inorganic lead halide perovskites as a potentially valuable and emerging materials for low-cost, high-efficiency SCs has been one of the most significant breakthroughs in the field of photovoltaics in recent years. Particularly, methylammonium-lead-trihalide perovskites (e.g., CH_3_NH_3_PbX_3_, where X = Cl, Br, or I) have been identified as potential next-generation competitive photoactive materials for applications in SCs.^494^ Due to significant improvements in the PCE, PSCs have gained attraction for building textile PSCs.^495^ Recently, a textile-based PSC has been developed employing low-temperature solution processing. The developed prototype of a planar PSC on a polyester/satin textile substrate demonstrates a PCE of 5.72%, along with considerable flexibility and durability under natural environmental conditions. Figure 16a shows a schematic of the fabricated PSC device, while Figure 16b illustrates the corresponding cross sectional SEM image of the developed PSC. Figure 16c shows the original photo of the fabricated device along with performance *J*_sc_ curve. Furthermore, in this research project the thickness of the perovskite layer and corresponding variation in performance was also observed and determined that the absorption spectral range shifts depend on the layer’s thickness.^458^ Recently, the configuration of Ti/c-TiO_2_/meso-TiO_2_/perovskite/spiro-OMeTAD/Au was transformed into a fiber structure to build a fiber shaped PSC. Under an AM 1.5 illumination, the fiber-shaped perovskite cells attained a PCE of 5.3%. Figure 16d and Figure 16e show the schematic layout and cross-sectional SEM image of the fabricated PSC, while Figure 16f presents the IV curve of the fabricated device, respectively.^496^ In another recent research study, a fiber-shaped PSC was developed by employing the configuration shown in Figure 16g. Figure 16h provides an illustration of the cross-sectional SEM image of the fabricated fiber-shaped PSC device. The corresponding PSC device achieved an improved PCE of 7.53%, reported by the same authors^496^ and a Voc of 0.96 V under an AM 1.5 G. illumination (Figure 16i).^205^ Recently, a flexible fiber-structured PSC was reported using stainless steel (SS) fiber as the working electrode and multiwall carbon nanotube (MWCNT) sheets as the counter electrode. This configuration obtained a maximum PCE of 3.3%.^200^ Another research group developed a fiber-shaped PSC using methylammonium lead iodide (CH_3_NH_3_PbI_3_) perovskite. The perovskite material was dipped in a solvent mixture of *N*,*N*-dimethylformamide (DMF) and *N*-methyl-2-pyrrolidone (NMP), followed by dipping in toluene. This approach achieved an impressive PCE of 3.85%.^497^ CNT-yarn-based perovskite SCs have also been recently developed. A layer of TiO_2_ oxide was formed on the twisting CNT yarns, which was then annealed using TiCl_4_ to establish a homogeneous ETL. A dip coating method was used to create a homogeneous perovskite layer on top of the TiO_2_ layer. A platinized carbon nanotube yarn was twisted around the top of the hole transport layer as the counter electrode. The developed yarn-based PSC device demonstrated a PCE of 0.63%, along with a high *V*_oc_ of 0.82 V.^498^Table 2 summarizes some of the recent work on textile and fiber-based PSCs.

### Textile-Based OSC Performance

OSCs have swiftly emerged as a leading option for the next-generation of flexible power sources as an emphasis has been focused on portable electronics. This is because of the unique qualities of OSCs, including their versatility, lightweight nature, low cost, and minimal impact on the environment.^499−501^ Notably, OSCs exhibit significantly better mechanical stability compared to other types of SCs, making them particularly suitable for wearable electronic textiles.^502−504^ Recent advancements have led to the development of lightweight and flexible organic SCs that maintain a constant level of PCE regardless of the direction of illumination.^505^ Recently, the active layer of the textile OSC was developed by using the dip-coating method, employing poly(3-hexylthiophene):phenyl-C61-butyric acid methyl ester (P3HT:PCBM) as the active material. In the developed OSC the substrate was made of Ti wires with perpendicularly aligned TiO_2_ nanotubes serving as the cathode, and CNTs with substantial electrical and mechanical properties were used as the anode. This textile-based OSC attained an efficiency of 1.08% and remained unaffected by mechanical bending for up to 200 cycles.^462^ Another research group developed a textile-based OSC that achieved a PCE of 2% and a current density of 13 mA/cm^2^. The textile-based OSC was fabricated employing P3HT:PCBM as an absorbing layer and a gold-based textile electrode served as the bottom electrode. Following fabrication, the developed textile-based OSCs were stitched onto a shirt and demonstrated stability with mechanical bending at a speed of 3 cm/s. Changes in the textile’s electrode resistivity with bending were observed but this was reversible in the reset position.^506^

**Figure 17:**
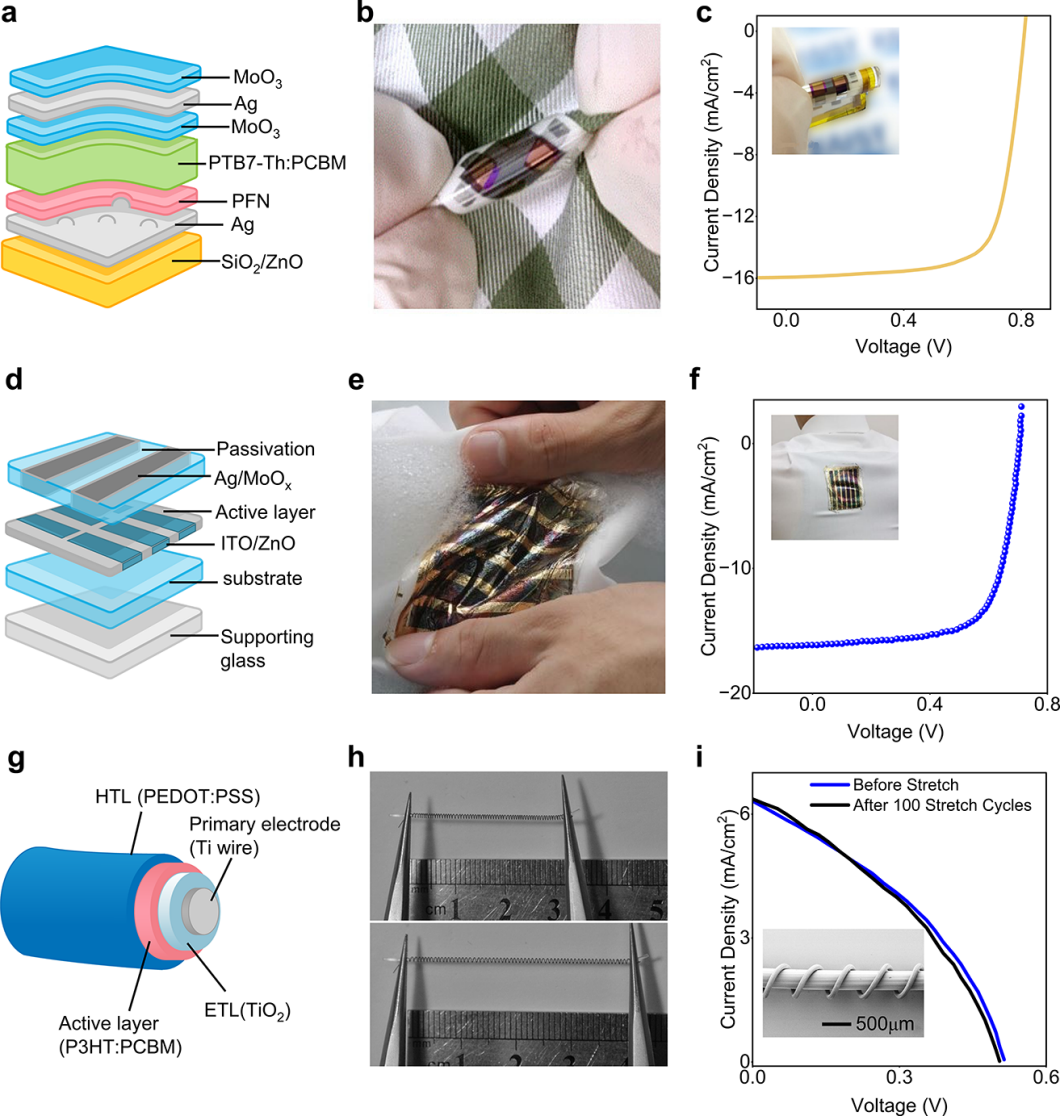
Performance evaluation for OSC photovoltaic textiles. (a) Schematic of a fabricated textile-based OSC, (b) a photograph of textile-based OSCs and (c) the IV curve for 100 bending cycles which is almost similar, demonstrating the flexibility of the fabricated device. Reproduced with permission from ref (131). Copyright 2019 American Chemical Society. (d) Schematic of a textile based OSC, (e) photograph of a device fixed on a shirt, showing high flexibility, and (f) the performance curve of the device in free stand mode on a perylene substrate. Reproduced with permission from ref (507). Copyright 2017 Springer Nature. (g) Schematic of fiber-shaped OSC, (h) a photograph of the fabricated fiber-shaped OSCs both before and after deformation, and (i) the current density curve along with an SEM image (inset). Reproduced with permission from ref (509). Copyright 2014 John Wiley and Sons.

A textile-based OSC was developed by incorporating plasmonic nanostructures onto commercially available woven fabrics that are typically incompatible with organic SCs due to their spatial opacity, irregularity, and physical porosity. The schematic configuration of the fabricated OSC is shown in Figure 17a. Spin coating was employed for the deposition of an active layer and electrodes. Figure 17b shows the photograph of the device integrated into the textile substrate, determining the flexibility of the textile-based OSC. Furthermore, the developed textile-based OSC achieved an impressive PCE of 8.71% (Figure 17c) and maintained its IV performance for more than 100 bending cycles.^131^Figure 17d presents the schematic of another recently investigated flexible and waterproof OSC that was coated with an elastomer. The device was attached to a shirt, as shown in Figure 17e, achieving a high PCE of 7.9%. Only a 5.4% reduction was observed after 2 h of water exposure. Figure 17f shows the IV curve of the OSC device when attached to a shirt.^507^

Fiber-shaped textile OSCs have recently gained attention due to their flexible nature.^508^ A spring-like organic SC has been developed to gain high stretchability and foldability, using Ti wire as the substrate, and by coating with TiO_2_ nanotubes, employing the electrochemical process (Figure 17g–i). The active material P3HT:PCBM and hole transport material PEDOT:PSS were subsequently coated. The device maintained a PCE of 90% even after more than 1000 bending cycles.^509^ However, these spring-type SCs exhibit strong mechanical stability but poor electrical responses due to limited exposure to sunlight. While mechanical stability and liability are important, the electrical performance of a SC, such as its PCE, is a crucial parameter metric to functionalize the perspective device in real-time applications. For further insights, Table 2 presents a summary of key research findings in textile-based OSCs. These results collectively indicate a promising future for the market of textile-based OSCs.

### Textile-Based QDSCs Performance

Recently, QDSCs have attracted considerable interest as a potential alternative to traditional SC technologies.^525^ QDs possess a tunable bandgap, which can be adjusted by varying their size.^526^ Notably, QDs have been incorporated into SCs, leading to significant improvements in both PCE and stability.^527^ For instance, the use of multication Cd_*x*_Zn_1–*x*_Se_*y*_S_1–*y*_ QDs as an interfacial modifying layer in a PSC device has resulted in reduced defects and traps, decreased recombination losses, enhanced electron extraction rates, and impressive PCE of 21.63%.^528^ In another study, researchers investigated the impact of different counter electrodes on QDSCs and found that a c-fabric/WO_3_-x electrode exhibited the highest power conversion efficiency among nine tested electrodes, reaching 4.6%.^529^ Despite the promising properties of QDs, their utilization in textile-based QDSCs has not received much attention due to concerns over their toxic nature. QDs commonly used, such as II–VI and IV–VI QDs, often contain heavy metal particles like cadmium (Cd), which are known to be highly toxic.^530,531^ Additionally, both Cd and selenium ions, present in the QD core, have recognized cytotoxic effects.^532^ Apart from toxic QDs, there have been researching reports of environment-friendly QDs being investigated for SCs applications.^533,534^ Ternary I–III–VI QDs, for example, possess favorable optical characteristics and are non-toxic. By incorporating these QDs into SCs, it is possible to improve the performance of SCs without introducing additional toxic components. Non-toxic (I–III–VI Copper indium sulfide (CuInS_2_)) QDs exhibit an excellent conduction bandgap (CB) and valence bandgap (VB) alignment with materials like MAPbI_3_ and graphene. Moreover, they exhibit high adsorption coefficients, leading to improved photoactivity and electron injecting. However, it should be noted that CuInS_2_ QDs are prone to air instability and have a low oxygen resistance.^535^ To further advance the field, it is necessary to develop stable encapsulation techniques that maintain performance while ensuring environmental sustainability. Further research on the exploration of sustainable and environmentally friendly QD materials is required to contribute to the positive potential of flexible and wearable solar energy harvesters.^530^

### Comparative Analysis of Third-Generation SCs

Continuing the exploration into the performance of third-generation SCs, a comprehensive comparative discussion becomes pivotal to delineate the future direction and scope for textile-based SCs. Table 3 presents a concise comparative analysis highlighting the strengths and weaknesses of each technology as applied to textile-based SCs. As observed from a diverse range of researchers’ perspectives, each technology exhibits its unique limitations and advantages, which should be considered in tandem with various parameters such as cost, durability, and efficiency. However, an overarching observation indicates several gaps are yet to be addressed in the domain of textile-based SCs before contemplating commercialization.

**Table 3:** Advantages and Disadvantages of Different Third-Generation SCs Technologies

SCs Technology	Advantages	Disadvantages	Ref
DSSCs	Easy fabrication, Flexibility, Versatility in low light condition, cost-effective	Electrolyte leakage and volatile, Stability concerns, Sensitive to low and high temperature, Less durable	(536−540)
PSCs	Rapid improvement in PCE, Low-cost fabrication, Solution Processable, Potential technology for high PCE to compete Si-based SCs	Less stable, Sensitivity to moisture, Sensitivity to UV light, Some are hazardous because of the inclusion of lead	(541−545)
OSCs	Flexibility, Solution processable, Lightweight, Low-cost materials	Lower efficiency, Poor stability and durability, Some materials are Oxidizing	(546−548)
QDSCs	Tunable bandgap, Low production cost, High efficiency, Multiple excitons generation capability, Solution processable	Some quantum dots are very toxic, Undesirable recombination, Low stability	(190, 532, 549)

## Wearable Properties of Textile-Based Solar Cells

Textiles have played a significant role in human society since the early stages of our evolution. Initially, textiles serve as a protective shelter against different weather conditions. However, as human civilization has progressed, textiles have transformed into garments and become an integral part of our daily lives. Recently, there has been a growing interest in the potential of energy-harvesting textiles in the field of wearable electronics. Various energy harvesting devices have been developed and integrated into textiles, and among them, SCs have emerged as a particularly promising option due to their affordability and widespread availability. The growing popularity of flexible SCs, such as third-generation SCs, has captured the attention of researchers in the field of electronic textiles.^38,550^ The emergence of textile-based SCs has expanded opportunities for scientists aiming to harvest solar energy without altering the inherent characteristics of textiles.^551^

### Comfortability, Breathability, Flexibility, and Appearance

When choosing a textile for a particular application, especially in the case of clothing, two crucial considerations are the garment’s comfort and overall appearance. E-textiles, on the other hand, integrate modules such as sensors, controllers, and so on, which make the textile more attractive as compared to ordinary textiles.^552,553^ The incorporation of SCs into textiles allows for efficient energy harvesting, but it disrupts their original state through the deposition of different materials via physical, chemical, and thermal processes. One of the key concerns in energy-harvesting textiles is finding a balance between maintaining their original characteristics, such as breathability, and avoiding stiffness and altered appearance resulting from the various processes involved.^554−556^ Recently, a significant advancement was made in the development of an OSC-based fabric, which achieved the practical implementation of OSC textiles at a meter-scale using an industrial loom. This innovative approach effectively combined device fabrication, textile weaving, and circuit connection into a single process, resulting in improved performance of OSC textiles accelerating their commercialization.^554^ However, there is still room for improvement in terms of appearance, comfort, and electrical performance to compete with the existing energy harvesting technologies.

Textiles are typically porous structures, to be breathable. However, the embedding of SCs into textiles can have an impact on their breathability. Specifically, the direct layer-by-layer fabrication of an interface and SC layers over textile surfaces can reduce the porosity and permeability of the fabric, hindering its ability to wick away moisture. Additionally, the heat transmission properties of the fabric may also be affected. A common approach involves affixing prefabricated OSCs to textiles, potentially resolving integration issues. However, ensuring a balance in flexibility between the SCs and the textile remains critical. Recent progress in flexible SCs has demonstrated high mechanical stability and exceptional stretchability,^557,558^ showing promise for seamless textile integration. Nevertheless, the attachment of these prefabricated devices may compromise the original state of the textile and impact the output quality due to the use of adhesive materials etc. Another potential solution to address this issue is the development of solar textiles using yarn intersections. This approach allows for the integration of SCs without compromising the breathability of the fabric. It is also important to consider fabrication techniques that involve processing at low temperatures, as high-temperature processing can distort the shape of the textile. Using curing materials that can be processed at room temperature, such as UV curable materials may offer a solution to maintain the breathability of the textiles while incorporating SCs.^216,559^

Contemplating the future of wearable textile-based SCs, significant strides can be made to enhance flexibility, wearability, and efficiency. A pivotal measure involves the development of flexible fabric electrodes tailored for optimal SC configurations, necessitating improvements in electrical conductivity, transmittance characteristics, surface smoothness, and water-resistant attributes. From a broader perspective, fibrous SCs exhibit distinct advantages over traditional flat SCs, being lightweight, easily manufacturable, wearable, and adaptable to curved surfaces such as the human body. However, a notable impediment to the widespread adoption of fibrous SCs is the potential presence of hazardous materials, limiting their suitability for wearable applications. For instance, the prevalent use of liquid electrolytes in fibrous DSSCs results in cumbersome device packaging and reduced stability. In contrast, fibrous PSCs and OSCs address these concerns by eliminating liquid components.^560^ Despite this favorable attribute, the commercialization of such textile-based SCs faces challenges related to output efficiency, stability, and durability, hindering their widespread market acceptance.

### Durability and Stability

Ensuring long-term reliability in both mechanical and electrical aspects is essential for bringing a product to market and competing with existing products. According to a press release published by ira.org,^561^ there is a forecast indicating a significant increase in worldwide renewable electricity production by over 60% from 2020 levels to exceed 4,800 GW by 2026. This would be comparable to the current total electricity production from fossil fuels and nuclear combined. Renewable energy generation is expected to contribute to approximately 95% of the global power capacity growth during this period, with solar energy harvesting accounting for more than half of this growth. Si-based SCs, which currently dominate the commercial market with a 90% share, are known for their robustness and reliability due to the sturdy substrates they employ, such as silicon wafers or glass. However, when it comes to integrating SCs into textiles, the various chemical and physical processes involved can disrupt the mechanical durability and overall stability of the textiles. Therefore, ensuring the long-term reliability and stability of textile-based SCs remains a challenge. Further research and advancements are needed to enhance the mechanical durability and stability of these textile-based systems to compete with the well-established Si-based SCs.

As an alternative to using hard conductive connections, a technique that has gained attention involves utilizing conductive carbon-based yarns as electrodes. Another option for improving textile-based SCs is to enhance their structure and configuration. For example, comb-shaped structures, have shown to be more adaptable compared to planar square structures, suggesting that further improvements in structure could potentially lead to optimal levels of both durability and stable performance. In summary, researchers in the field need to consider the longevity and stable output of Textile-based SCs, as their electrical performance tends to degrade over time.^562^ Therefore, exploring innovative approaches and refining the design and construction of Textile-based SCs can contribute to achieving long-lasting and reliable performance.

### Washability

**Figure 18:**
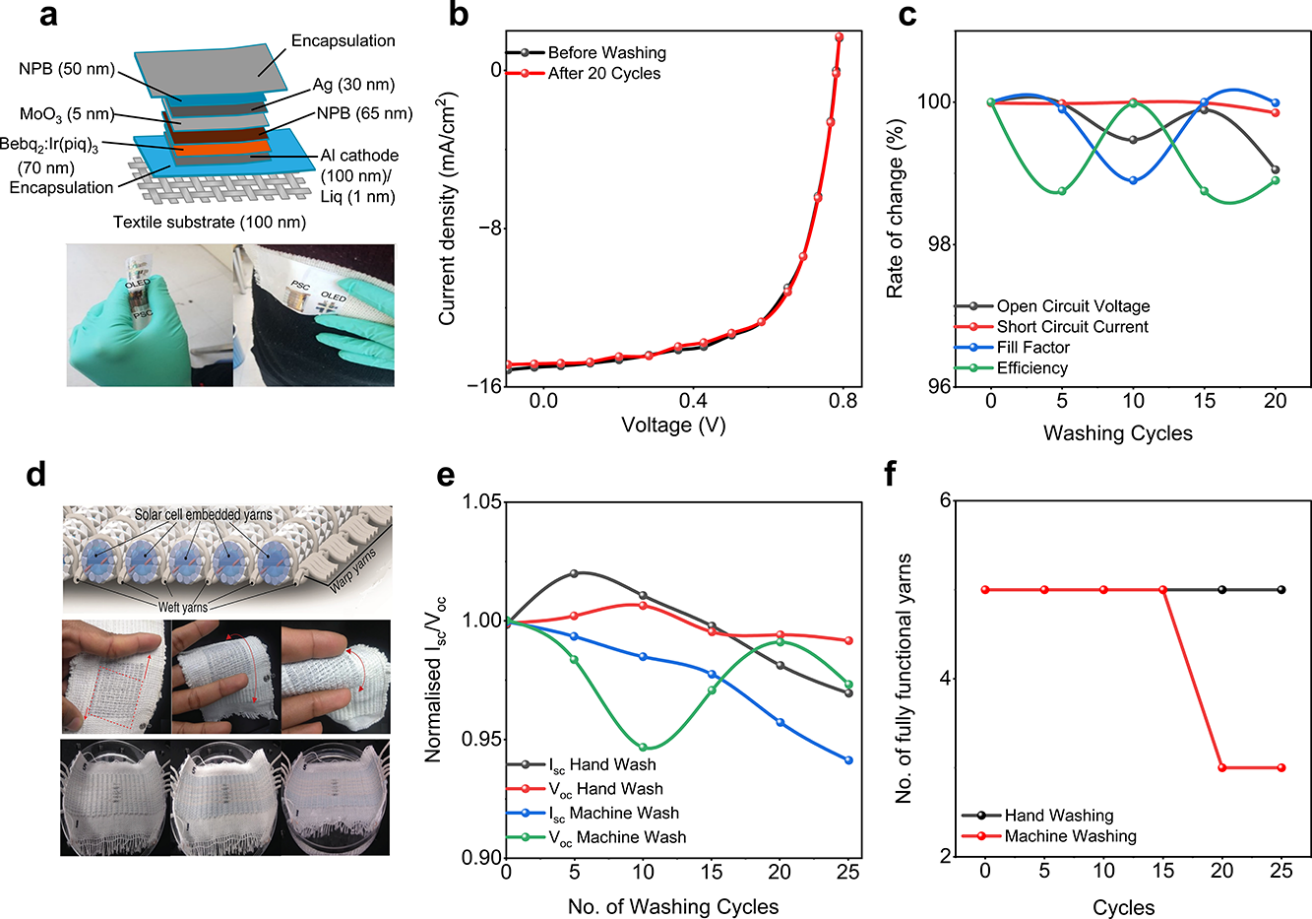
Washability performance evaluation for photovoltaic textiles. (a) Photographs of a fabricated textile-based OSC device, (b) electrical performance curves after passing through different washing cycles and (c) the demonstration of the relative changes in performance with respect to washing cycles such as *V*_oc_, PCE. Reproduced with permission from ref (563). Copyright 2018 The Royal Society of Chemistry. (d) In these set of images, five solar-E-yarns woven into a textile are shown in their dry state, soaked with tap water, and immersed in tap water. (e) The normalized *I*_SC_ with different hand and machine-washing cycles, and (f) the number of fully functional solar-E-yarns after 25 washing cycles. Reproduced with permission under a Creative Commons CC-BY License from ref (130). Copyright 2019 John Wiley and Sons.

The ability of a textile to withstand multiple washes and drying cycles is crucial for its usability as a wearable item. Wearable e-textiles are designed to maintain their attractiveness or original form over time, as this is their primary function. However, the incorporation of SCs into fabrics introduces a sensitivity to cleaning agents, as there has been limited advancement in making textile-based SCs resistant to mechanical or chemical washing. Researchers have been exploring various materials and processes to improve the washability of textile-based SCs without compromising their performance. For instance, coating methods have been employed to develop textile-based OSCs that have undergone more than 20 washing cycles. Figure 18a depicts a photograph of a textile-based OSC, while Figure 18b and c illustrate the impact on performance after various washing (both hand and machine) and bending cycles. Regardless of the number of washing cycles, the device attained a PCE of up to 7.24%.^563^

Another research team conducted a study where they developed a solar fabric with SCs integrated into yarns and subjected it to hand and machine-washing cycles. Figure 18d presents photographs of a fabric in dry, wet, and immersed conditions, from right to left. Figure 18e showcases the corresponding *I*_sc_ values, indicating that the *I*_sc_ values were even better than those observed under dry conditions. Figure 18f demonstrates that all SC yarns remained functional up to 15 washing cycles, regardless of whether it was hand or machine washed.^130^ However, still there is huge room for research investigation to make washable textile-based SCs without any loss for commercial applications.

### Safety and Toxicity

The integration of SCs involves the use of many hazardous chemicals. For instance, the active perovskite materials in PSC, dyes in DSSCs, and thin film materials such as CdTe are known to be hazardous to the skin and can cause serious effects upon direct interaction.^564^ While textiles and garments are typically designed to have no adverse effect on human skin, the integration of SCs introduces these potentially harmful chemicals. DSSCs, due to their high efficiency, are being used in textile-based SCs; however, there are concerns regarding evaporation and leakage of liquid dye, which could lead to complications for the skin and other tissues. To meet the requirements of future textile-based DSSC applications, it is necessary to use inexpensive and environmentally friendly materials, as well as improve the safety of the dyes. Researchers have been exploring nontoxic solutions, such as using dimethyl sulfoxide (DMSO) as a nontoxic solvent for gel electrolytes that do not include ionic liquids.^565^ If textile-based SCs are to be used in clothing, extreme caution must be exercised due to direct contact with the human body. Implementing proper isolation from the surroundings, adequate packaging to minimize ecological impacts, and ensuring electric shock/spark-free systems are crucial criteria to avoid any risk. Consequently, research and development efforts in textile-based SCs prioritize correct packaging and the selection of materials with reduced or no risk.

## Future Research Direction of Textile-Based Solar Cells

**Figure 19:**
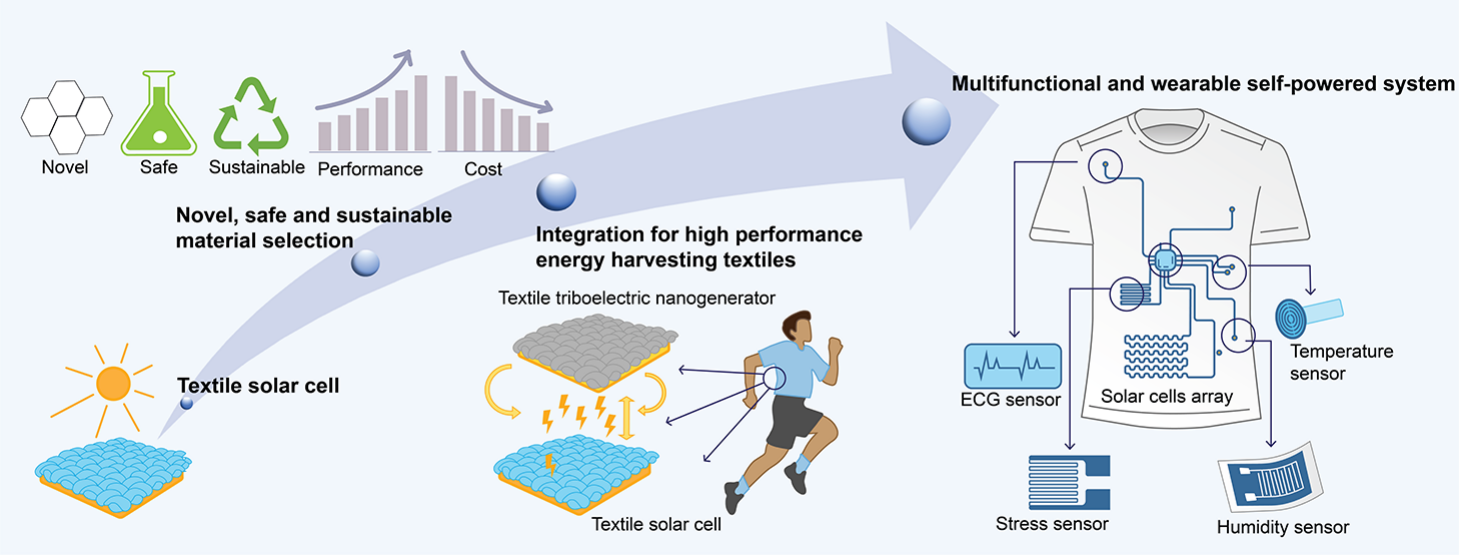
Future perspectives of smart photovoltaic textiles.

According to research conducted by Global Industry Analysts Inc. (2022),^566^ the global market for smart textiles is expected to reach $5.9 billion by 2026. Sectors such as sports and wellness, healthcare, safety, and industrial workwear show great potential for the utilization of smart e-textiles. However, for wearable technology to function effectively, it requires a reliable source of electrical energy. As early as 2010, researchers started exploring the possibility of integrating SCs into fabrics.^487,567^ SCs have been incorporated into various products like bags, tents, and helmets, to provide energy for electronics. However, the design options for textiles with attached SCs are limited because the SCs are visible and occupy a significant portion of the product’s surface. Although incorporating SCs into yarns may have aesthetic advantages, their limited surface coverage reduces the amount of power generated per unit area.^109^ However, the production of SCs directly in parallel with textile manufacturing, creating a fully textile-based energy harvester, requires additional efforts to ensure flexibility and efficiency.^294,495,552^ The ongoing research on textile-based energy harvesting SCs indicates a strong potential for a significant commercial market for such products in the future. A general perspective for future wearable textiles is illustrated in Figure 19.

### Toward High Performance Energy Harvesting Textiles

The current reported efficiency of c-SCs is only a maximum of 26.7% on a rigid substrate such as a silicon wafer,^137^ indicating that there is significant room for improvement. First-generation SCs, as previously mentioned, are constructed on stiff surfaces, and require high-temperature processing, making them unsuitable for fabrics and flexible substrates. While second-generation SCs are thinner than first-generation cells, many of the thin film materials are toxic (such as CdTe) and rare in terms of availability.^568,569^ Therefore, the focus is now shifting toward third-generation SCs technology, which emphasizes lightweight and flexible structures that can be processed at low temperatures. This makes them a primary consideration for the development of next-generation textile-based SCs. The choice of electrodes directly impacts the performance of SCs. The use of metallic or rigid electrodes is not preferable for textile-based SCs due to their stiffness, which also affects breathability.^568,569^ To address this, newly developed conductive materials that are suitable for textiles and possess good conductivity can improve the output response of textile-based SCs (see Figure 19). For instance, replacing metallic and rigid electrodes with carbon and polymer-based electrodes^339,340,570^ can potentially enhance the overall PCE of the textile-based SCs. Additionally, the choice of materials for charge transport layers and active materials is crucial and warrants further research for improvement. One example is the utilization of composite materials such as P3HT in combination with other materials like PCBM,^571−573^ among others. Developing SCs on a rough surface such as textile can lead to the degradation of the deposited layers, resulting in cracks and void defects. These defects act as barriers to spectral absorption and proper charge transportation. To address this, it is possible to smooth the surface of textiles by utilizing interface materials. Researchers have employed interface layers like UV curable polyurethane (PU), polyamide,^476^ and others to achieve a smoother surface for the fine deposition of different layers via printing or coating. However, it is important to consider that using such interface materials may affect the original state and uniqueness of the textiles.^476^ The selection of encapsulation materials that are both flexible and robust, with reduced thickness, may prove more effective than bulky materials. Another approach to enhancing the performance of textile-based SCs is by combining them with other renewable energy harvesters, such as piezoelectric nanogenerators (PENG), triboelectric nanogenerators (TENG), and thermoelectric nanogenerators (TNG). These types of energy harvesters can produce significant amounts of electricity. However, their durability has been a limiting factor for commercial applications. Therefore, combining the effects of various nanogenerators has garnered attention to generate more power, as shown in Figure 19.^55^

### Toward Sustainable Energy Solutions to Wearables

The rapid development of the electronic industry has led to an increased demand for high-performance portable and wearable power supply units. However, with the growing focus on energy consumption and environmental protection worldwide, there is a rising interest in using clean energy sources.^324^ As a result, it has become imperative to explore safe and sustainable manufacturing methods for energy harvesting devices.^553^ The development of new eco-friendly and cost-efficient energy generation systems is crucial to address emerging ecological concerns and meet the needs of modern society.^361^Figure 19 also illustrates the concept of sustainability in energy harvesting SCs, often referred to as “green energy”. This concept involves extending the lifespan of the device, reducing costs, and most importantly, ensuring recyclability.^361^ Sunlight is the most abundant source of clean energy available to us; however, the process of harnessing solar energy through SCs may pose health risks when used in wearables, primarily due to the presence of hazardous materials such as CdTe, perovskites (containing lead halides), and other functional materials. Sustainable, scalable, plentiful, renewable, and environmentally friendly energy generation using biomaterials is the ultimate goal of clean energy harvesting.^574^ Generating electric power from sunlight through SCs has the potential to become a widespread, biodegradable energy source that is also commercially viable.^575^ Further research into textile-based SCs is necessary, particularly in exploring electrode materials that are less harmful, biodegradable, and recyclable. The selection of sustainable materials for electrodes and other functional layers is a critical factor that directly impacts the performance of SCs. Additionally, choosing appropriate encapsulation materials that can stop the migration of any hazardous materials is also important. However, the primary consideration of any industry’s sustainability is improving product performance while reducing production costs, and improving recyclability;the same applies to the SCs industry. Alongside improving manufacturing processes and technologies, identifying stable and effective biodegradable materials while reducing cost is a focus of future research. Substituting existing materials with low-cost raw materials, such as natural mineral resources, could be an attractive option. Furthermore, combining low-cost raw materials with high-priced ones without compromising performance could be another approach to reduce the overall cost of SCs.

### Toward Multifunctional Wearable Self-Powered Systems

Due to the advancements in electronics miniaturization, nanotechnology and the digital revolution, smart wearable e-textiles have made significant progress in the past decade. Such advancements in flexible and wearable technologies have enabled the creation of customized wearable textiles capable of interacting with the body, continuously monitoring, recording, and communicating various physiological information.^361^Figure 19 presents a schematic of a multifunctional garment that incorporates basic physiological parameters of the human body. E-textile-based sensors for electrocardiogram (ECG),^576^ and electroencephalogram (EEG),^466^ strain sensors for electrophysiological signal sensing,^465^ body-temperature sensors,^577^ humidity sensors,^578^ and others have already been reported. However, there are still many challenges to overcome in integrating them into a self-powered multifunctional system. For instance, a separate power supply source is required, as well as additional circuitry for transferring body signals. E-textiles with a single functionality are insufficient to meet the demands of modern electronics. Therefore, increasing attention is focused on achieving functional integration among energy generation, storage, and utilization to power multiple functionalities within a single e-textile.^361,466^ Various strategies can be employed to accomplish this goal. One approach is the development of specialized yarns for various purposes, including energy harvesting yarns, energy storage yarns, and various sensor-based yarns, all woven to create a multifunctional e-textile. Another potential solution lies in the selection of functional materials that can be applied across different applications. For example, ZnO possess characteristics that make it suitable for use as stress and heat sensors, allowing it to provide dual benefits. Additionally, textiles composed of multiple thin layers, with each layer serving a different purpose such as energy harvesting, storage, and sensing, could be a viable solution. However, overcoming these existing challenges and achieving fully multifunctional,^579^ self-powered textiles that are suitable for commercialization will require significant effort. In the future, integrating SCs with sensors, actuators, electrochromic, shape memory, and even self-repair capabilities will be highly appealing for the development of multifunctional and self-powered personalized healthcare textiles.^560^

## Conclusions

Portable and wearable e-textiles have garnered significant attention due to their wide range of applications in healthcare, military, entertainment, among others. This increasing demand has resulted in the growth in the popularity of methods that produce electricity energy from solar energy conversion. The development of wearable e-textiles capable of generating electricity holds great potential in the energy and wearable sectors, especially in the era of the Internet of Things and modern communication. It is worth giving a timely summary of the remarkable progress in the community of textile-based solar cells, which could promote their industrial commercialization and being in accordance with the standards of the photovoltaic market in the near future.

## Vocabulary

energy harvesting: process of converting ambient energy into useful electrical power
photovoltaic effect: process of producing electrical power from sunlight in solar cells
solar cell: device that uses the photovoltaic effect in semiconductor materials to convert sunlight into electricity;
magnetoelastic generator: small device that converts ambient mechanical energy into electrical power based on magnetoelastic effect; nanogenerator: small device that converts mechanical energy into electrical power based on piezoelectric and trioelectric effects
wearable electronics: lightweight flexible devices that are worn on the body and incorporate technology for tasks such as health tracking, communication, etc.
electronic textile (e-textile): fabric or textile-based materials that are combined with electronic components, allowing the development of wearable technology or smart clothes that can perceive, respond, or interact with their surroundings or their wearer.
